# Supplementary material for: Thyrotropin-releasing hormone neurons of different hypothalamic nuclei increase energy expenditure
Source: Nat Commun. 2026 Apr 15;17:3499. doi: 10.1038/s41467-026-71617-3 (PMC13083850; doi:10.1038/s41467-026-71617-3)
Supplement: Supplementary file 1 — Supplementary Information [file 41467_2026_71617_MOESM1_ESM.pdf]

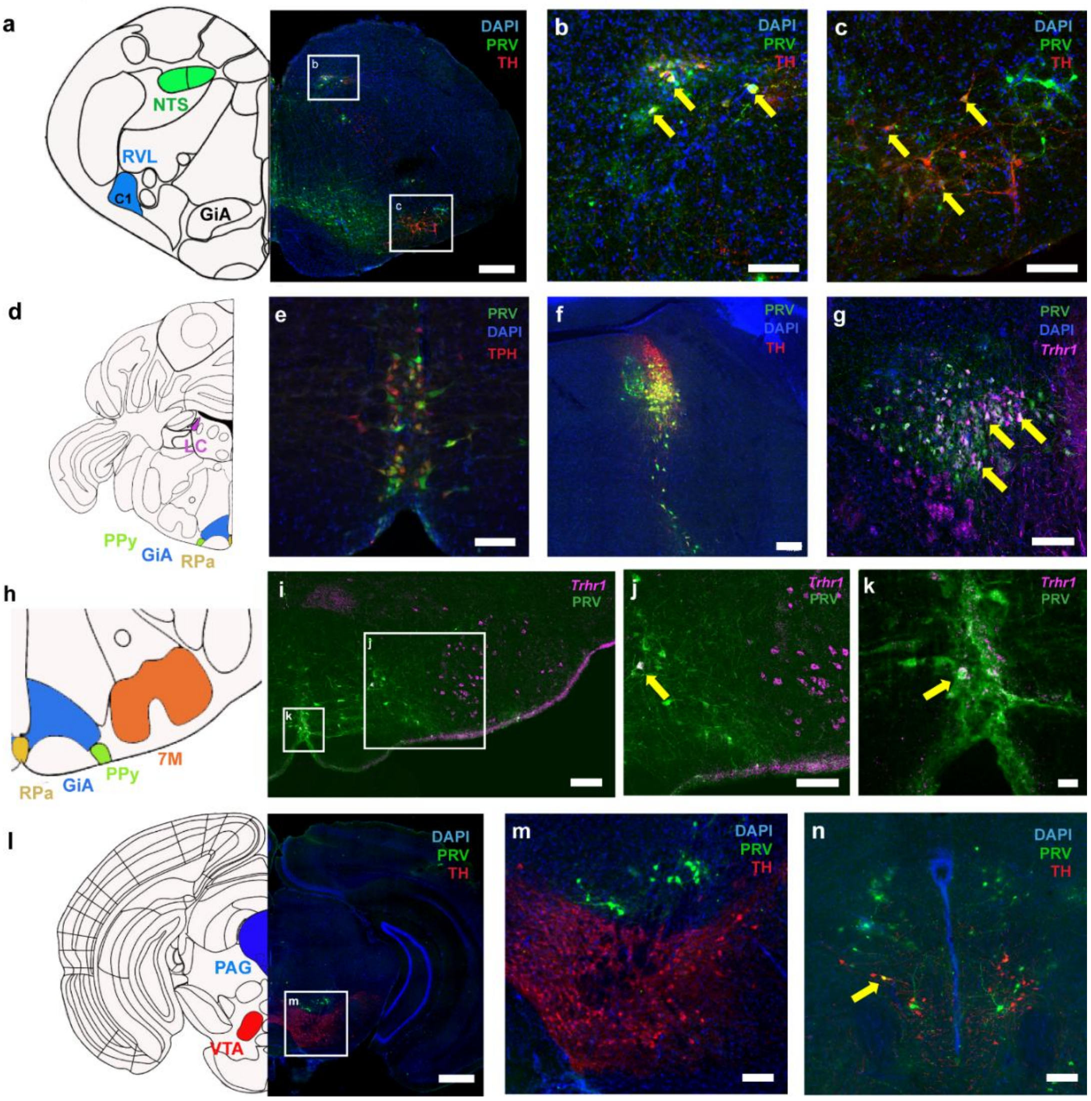


## Supplementary Figure 1

**Neuron populations that are polysynaptically connected to the BAT. a**) PRV positive cells (green) in the medulla oblongata in the area of the nucleus tractus solitarii (NTS) five days after injection of a GFP-expressing PRV into the BAT. **b-c**) Co-staining of PRV (green) positive cells located in the NTS (**b**) and the C1 area of the rostral ventrolateral medulla oblongata (C1 RVL, **c**) with the tyrosine hydroxylase (TH, red). **d**) Schematic drawing of the hindbrain in the area of the LC. **e**) Colocalization of PRV-positive cells in the RPa with tryptophan hydroxylase (TPH, red). **f**-**g**) Colocalization of PRV-positive cells of the LC with TH (**f**) and *Trhr1* mRNA (**g**). **h**) Schematic drawing of the mediobasal hindbrain in the area of the LC. **i-k**) Colocalizations of PRV positive cells (green) with *Trhr1* mRNA (magenta) in basal hindbrain with zoom-in for the border region between the gigantocellular reticular nucleus (GiA) and facial nucleus (7M, **j**) and the RPa (**k**). **l**) Schematic drawing of the midbrain in the area of the ventral tegmental area (VTA) showing the location of PRV (green)-positive cells costained with TH (red). Zoom in of the VTA (**m**) and the PAG (**n**). Colocalizations are indicated by an arrow. Scalebars: **a** and **l**: 500µm; **f** and **i**: 150µm; **b**, **c**, **e**, **g**, **j**, **m**, and **n**: 100µm; **k**: 20 µm. All staining and RNAScops were repeated at least 2 times in 3 independent animals. Drawings in a, d, h, and l were adapted from the Allen Reference Atlas – Mouse Brain [Adult Mouse]. Available from atlas.brain-map.org[1].

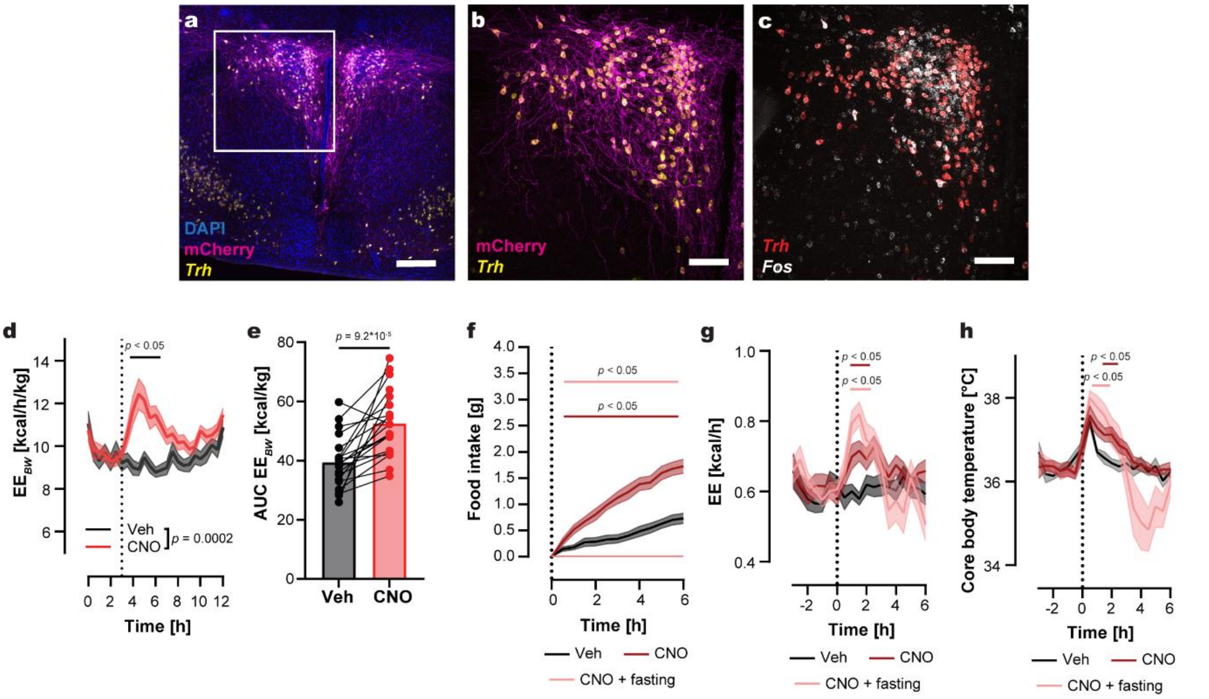


## Supplementary Figure 2

**Validation of PVN$^{TRH}$-hM3D mice. a**) Validation of the specificity of hM3D-mCherry expression in TRH neurons 3 weeks after AAV-CAG-flex(hM3D-mCherry) injection into the PVN of TRH-IRES-Cre mice. Immunohistochemistry against mCherry (magenta) combined with RNAScope against *Trh* (yellow). Nuclei were stained by DAPI (blue), scale bar: 250 µm. **b**) Zoom in of the marked area in a. Scale bar: 100 µm. **c**) RNAScope against *Fos* (white) and *Trh* (red) of the marked area in a 90 min after CNO injection. Scale bar: 100 µm. All stainings and RNAScops were repeated in 3 independent animals. **d**) Changes in EE normalized by body weight (EE$_{BW}$) after CNO (red) and vehicle (gray) treatment over time in PVN$^{TRH}$-hM3D mice. **e**) Individual changes of EE$_{BW}$ between vehicle and CNO stimulation shown as AUC of EE$_{BW}$ curves from d. **f-h**) Food intake (**f**), EE (**g**) and core body temperature (**h**) of PVN$^{TRH}$-hM3D mice treated with vehicle (gray), CNO (dark red) and CNO under food withdrawal (rose). All data presented are shown as mean ± s.e.m. of the biological replicates for each group. Animal-specific changes under vehicle compared to CNO treatment are indicated by connected data points. For b, two-tailed unpaired t-tests; for a, c, and d two-way RM ANOVA followed by Šídák's multiple-comparisons test; for e, a REML mixed-effects model followed by Šídák's multiple-comparisons test was used. Exact *p*-values, sample sizes and statistical details are provided in Supplementary Table 1.

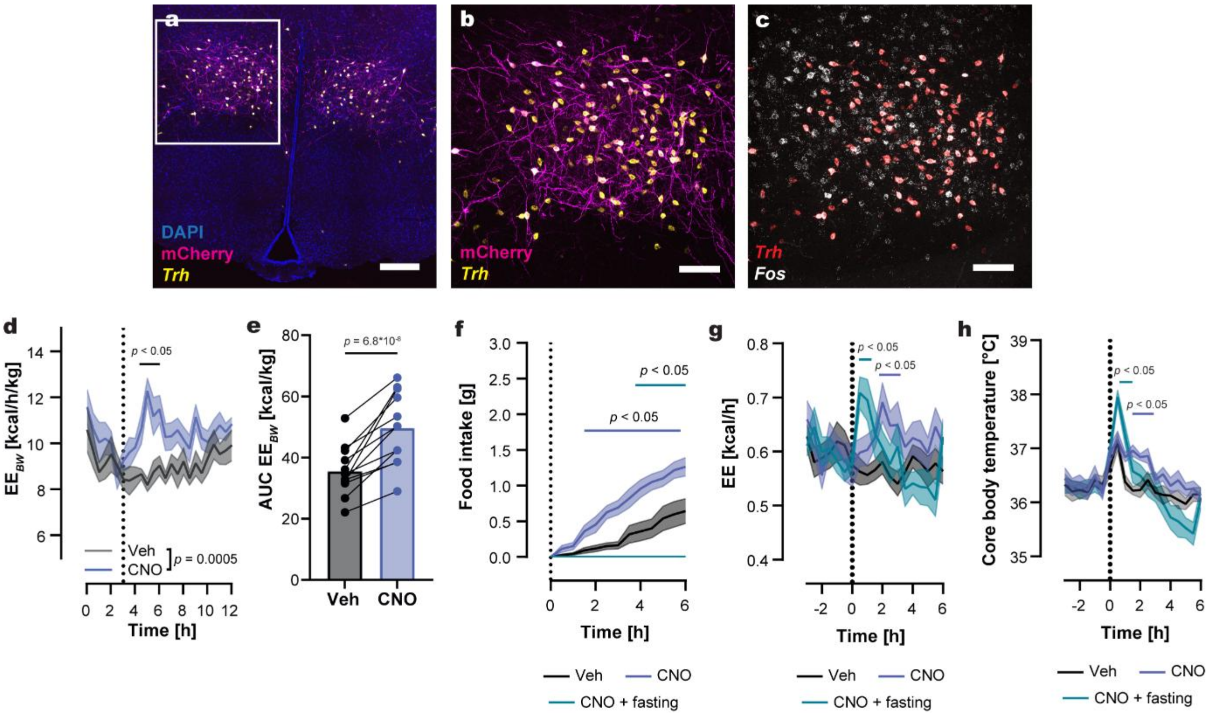


## Supplementary Figure 3

**Validation of DMH$^{TRH}$-hM3D mice. a**) Validation of the specificity of hM3D-mCherry expression in TRH neurons 3 weeks after AAV-CAG-flex(hM3D-mCherry) injection into the DMH of TRH-IRES-Cre mice. Immunohistochemistry against mCherry (magenta) combined with RNAScope against *Trh* (yellow). Nuclei were stained by DAPI (blue), scale bar: 250 µm. **b**) Zoom in of the marked area in a. Scale bar: 100 µm. **c**) RNAScope against *Fos* (white) and *Trh* (red) of the marked area in a 90 min after CNO injection. Scale bar: 100 µm. All staining and RNAScops were repeated in 3 independent animals. **d**) Changes in EE normalized by body weight ($EE_{BW}$) after CNO (blue) and vehicle (gray) treatment over time in DMH$^{TRH}$-hM3D mice. **e**) Individual changes of $EE_{BW}$ between vehicle and CNO stimulation shown as AUC of $EE_{BW}$ curves from d. **f-h**) Food intake (**f**), EE (**g**) and core body temperature (**h**) of DMH$^{TRH}$-hM3D mice treated with vehicle (gray), CNO (dark blue) and CNO under food withdrawal (light blue). All data presented are shown as mean ± s.e.m. of the biological replicates for each group. Animal-specific changes under vehicle compared to CNO treatment are indicated by connected data points. For b, two-tailed unpaired t-tests; for a, c, d and e two-way RM ANOVA followed by Šídák's multiple-comparisons test was used. Exact *p*-values, sample sizes and statistical details are provided in Supplementary Table 1.

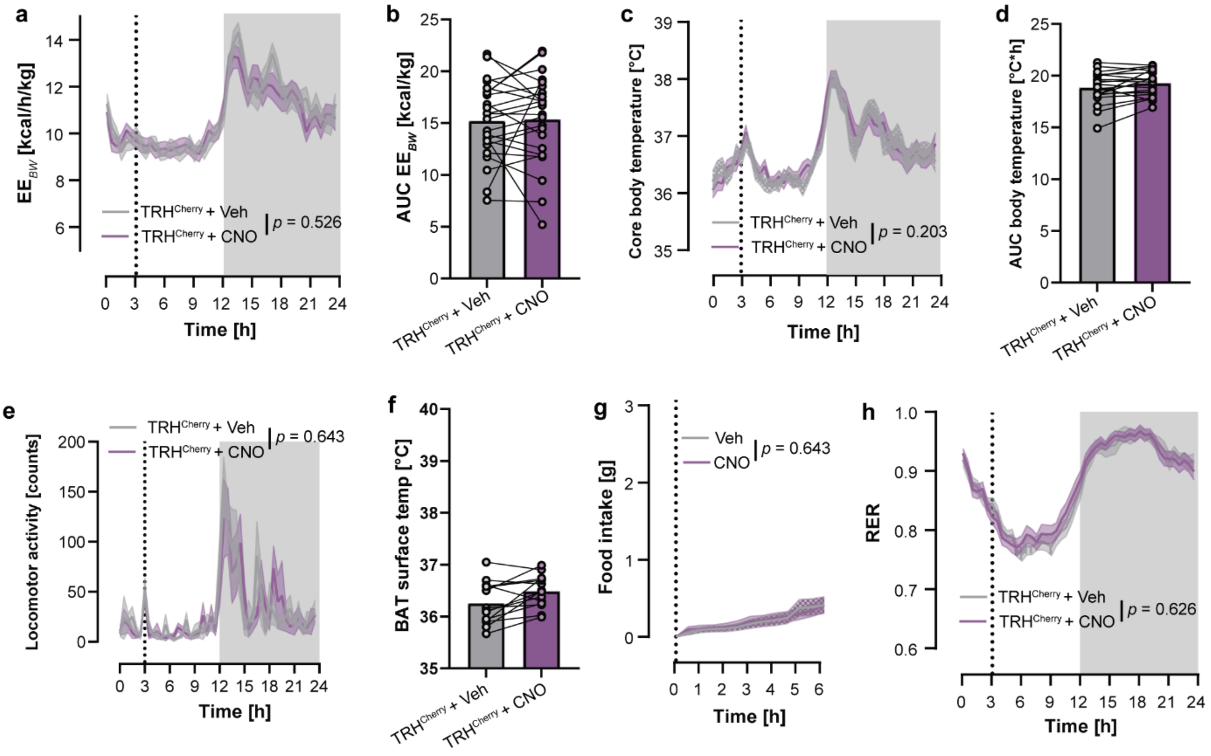


## Supplementary Figure 4

**Validation of TRH$^{Cherry}$ mice. a**) Changes in energy expenditure (EE) after CNO and vehicle treatment over time of TRH$^{Cherry}$ control mice. **b**) Changes of individual EE shown as AUC of EE curves from a. **c**) Changes in core body temperature after CNO and vehicle stimulation over time. **d**) Individual changes of core body temperature after vehicle and CNO stimulation shown as AUC of temperature curves from c. **e**) Changes in home cage activity after CNO and vehicle treatment over time. **f**) Animal individual changes in BAT surface temperature before (gray) and after treatment with CNO (purple) detected by IR-thermography. **g**) Cumulative food intake in mice treated with vehicle and CNO. **h**) RER curves of mice treated with CNO or vehicle during the light phase. All data presented in **a**, **c**, **e**, **g**, and **h** are shown as mean ± s.e.m. of the biological replicates for each group. Animal-specific changes under vehicle compared to CNO treatment are indicated by connected data points. For b, d, and f two-tailed unpaired t-tests; for a, c, e, g, and h a two-way RM ANOVA followed by Šídák's multiple-comparisons test was used. Exact *p*-values, sample sizes and statistical details are provided in Supplementary Table 1.

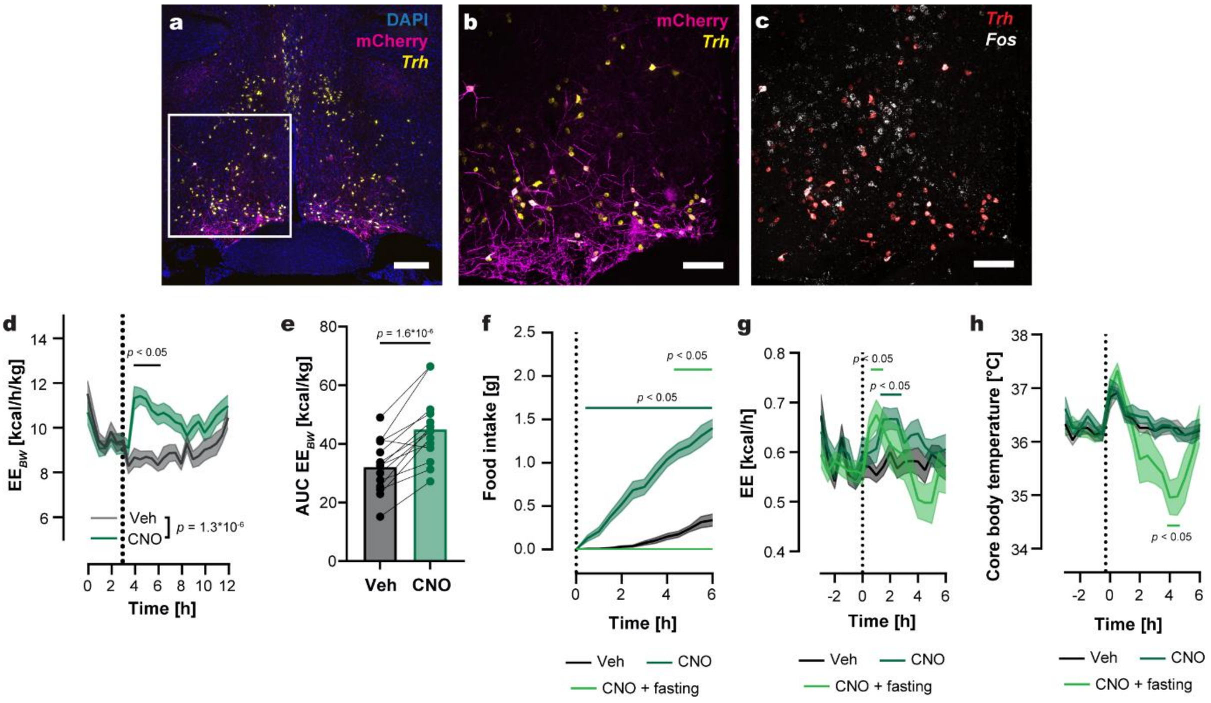


## Supplementary Figure 5

**Validation of $MPA^{TRH}$-hM3D mice. a**) Validation of the specificity of hM3D-mCherry expression in TRH neurons 3 weeks after AAV-CAG-flex(hM3D-mCherry) injection into the MPA of TRH-IRES-Cre mice. Immunohistochemistry against mCherry (magenta) combined with RNAScope against *Trh* (yellow). Nuclei were stained using DAPI (blue). Scale bar: 250 µm. **b**) Zoom in of the marked area in a. Scale bar: 100 µm. **c**) RNAScope against *Fos* (white) and *Trh* (red) of the marked area in a 90 min after CNO injection. Scale bar: 100 µm. All staining and RNAScops were repeated in 3 independent animals. **d**) Changes in EE normalized by body weight ($EE_{BW}$) after CNO (green) and vehicle (gray) treatment over time in $DMH^{TRH}$-hM3D mice. **e**) Individual changes of $EE_{BW}$ between vehicle and CNO stimulation shown as AUC of $EE_{BW}$ curves from d. **f-h**) Food intake (**f**), EE (**g**) and core body temperature (**h**) of $MPA^{TRH}$-hM3D mice treated with vehicle (gray), CNO (dark green) and CNO under food withdrawal (light green). All data presented are shown as mean ± s.e.m. of the biological replicates for each group. Animal-specific changes under vehicle compared to CNO treatment are indicated by connected data points. Animal-specific changes under vehicle compared to CNO treatment are indicated by connected data points. For b, a two-tailed unpaired t-tests; for a, c, d and e a two-way RM ANOVA followed by Šídák's multiple-comparisons test was used. Exact *p*-values, sample sizes and statistical details are provided in Supplementary Table 1.

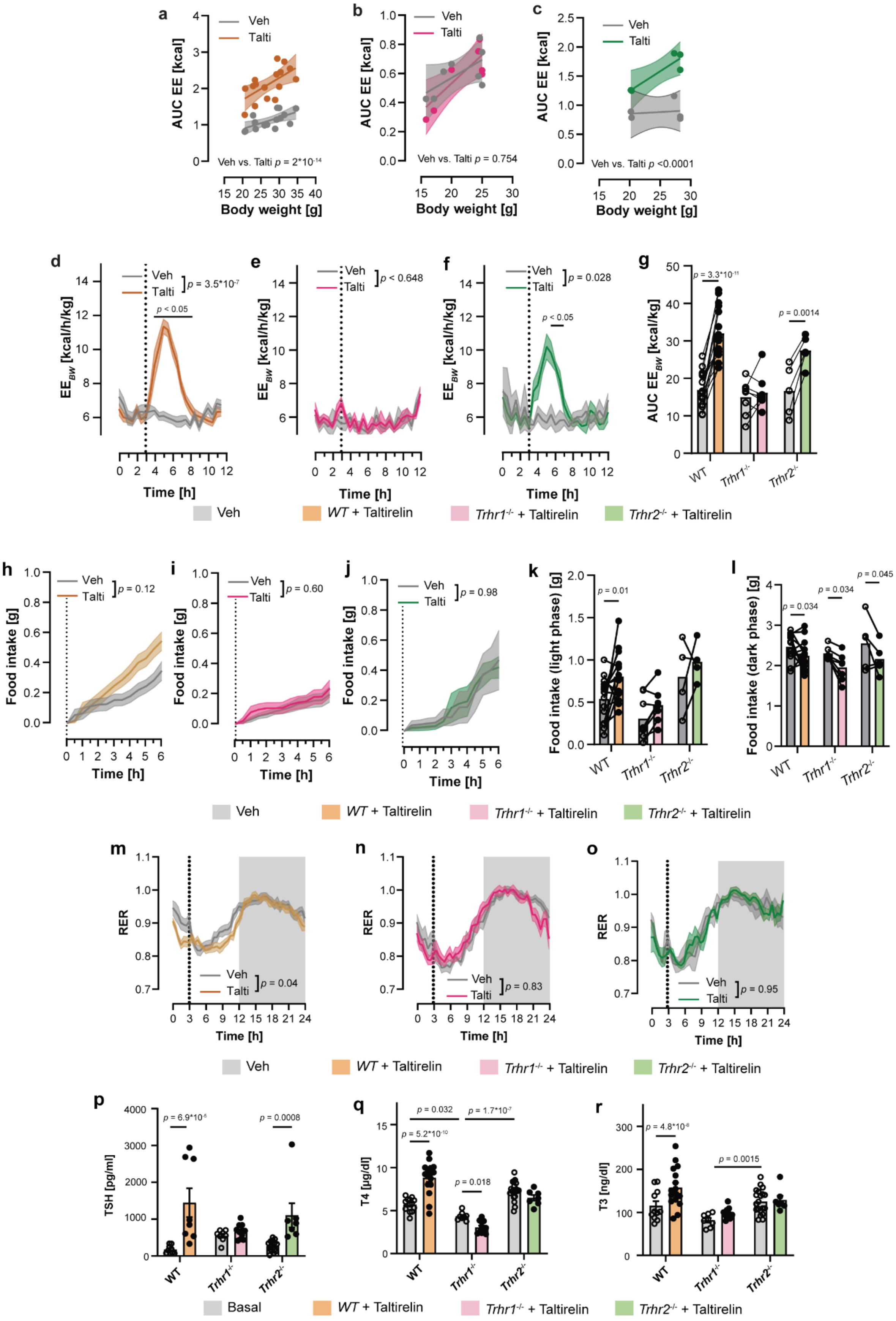
a
Veh
Talti
AUC EE [kcal]
Veh vs. Talti p = 2*10-14
Body weight [g]
b
Veh
Talti
AUC EE [kcal]
Veh vs. Talti p = 0.754
Body weight [g]
c
Veh
Talti
AUC EE [kcal]
Veh vs. Talti p <0.0001
Body weight [g]
d
Veh
Talti
p = 3.5*10-7
p < 0.05
EEBW [kcal/h/kg]
Time [h]
e
Veh
Talti
p < 0.648
EEBW [kcal/h/kg]
Time [h]
f
Veh
Talti
p = 0.028
p < 0.05
EEBW [kcal/h/kg]
Time [h]
g
p = 3.3*10-11
p = 0.0014
AUC EEBW [kcal/kg]
WT
Trhr1-/-
Trhr2-/-
Veh
WT + Taltirelin
Trhr1-/- + Taltirelin
Trhr2-/- + Taltirelin
h
Veh
Talti
p = 0.12
Food intake [g]
Time [h]
i
Veh
Talti
p = 0.60
Food intake [g]
Time [h]
j
Veh
Talti
p = 0.98
Food intake [g]
Time [h]
k
p = 0.01
Food intake (light phase) [g]
WT
Trhr1-/-
Trhr2-/-
l
p = 0.034
p = 0.034
p = 0.045
Food intake (dark phase) [g]
WT
Trhr1-/-
Trhr2-/-
Veh
WT + Taltirelin
Trhr1-/- + Taltirelin
Trhr2-/- + Taltirelin
m
RER
Veh
Talti
p = 0.04
Time [h]
n
RER
Veh
Talti
p = 0.83
Time [h]
o
RER
Veh
Talti
p = 0.95
Time [h]
Veh
WT + Taltirelin
Trhr1-/- + Taltirelin
Trhr2-/- + Taltirelin
p
p = 6.9*10-5
p = 0.0008
TSH [pg/ml]
WT
Trhr1-/-
Trhr2-/-
q
p = 0.032
p = 1.7*10-7
p = 5.2*10-10
p = 0.018
T4 [µg/dl]
WT
Trhr1-/-
Trhr2-/-
r
p = 4.8*10-6
p = 0.0015
T3 [ng/dl]
WT
Trhr1-/-
Trhr2-/-
Basal
WT + Taltirelin
Trhr1-/- + Taltirelin
Trhr2-/- + Taltirelin

## Supplementary Figure 6

**Validation of taltirelin effects. a-c**) Linear regression of the individual changes of the AUC EE from Fig. 6d-e against the body weight after vehicle (gray) and taltirelin treatment in *WT* (**a**, brown), *Trhr1*$^{-/-}$ (**b**, pink), and *Trhr2*$^{-/-}$ (**c**, green) mice for ANCOVA analysis. **d-f**) Changes in energy expenditure normalized by body weight ($EE_{BW}$) after vehicle (0.9 % NaCl, gray) or taltirelin treatment (1 $mg/kg_{bw}$, i.p., colored lines) over time in *WT* (**d**, brown), *Trhr1*$^{-/-}$ (**e**, pink), and *Trhr2*$^{-/-}$ (**f**, green) mice. The dotted line indicates injection time (i.p.) in a crossover design on two consecutive days. **g**) Individual changes in EE between vehicle and taltirelin treatment are shown as AUC of EE curves from d-f. **h-j**) Cumulative food intake over 6 h after vehicle (gray, 0.9 % NaCl) and taltirelin treatment (1 $mg/kg_{bw}$, i.p., colored line). *WT* (**h**, brown), *Trhr1*$^{-/-}$ (**i**, pink), and *Trhr2*$^{-/-}$ (**j**, green) mice were treated three hours after the light phase started in a crossover design on consecutive days. **k-l**) Quantification of individual changes in total food intake in the light (**k**) and dark (**l**) phase after vehicle (gray bars) or taltirelin treatment (colored bars). **m-o**) RER curves of *WT* (**m**, brown), *Trhr1*$^{-/-}$ (**n**, pink), and *Trhr2*$^{-/-}$ (**o**, green) mice treated with taltirelin (colored line) and vehicle (gray line). **p-r**) Changes of plasma TSH (**p**), T4 (**q**), and T3 (**r**) levels 90 min after stimulation with taltirelin (1 $mg/kg_{bw}$, i.p., colored bar) versus vehicle (0.9 % NaCl, i.p., gray bar) of *WT* (brown) *Trhr1*$^{-/-}$ (pink) or *Trhr2*$^{-/-}$(green) mice. All presented data are shown as mean ± s.e.m. of the biological replicates for each group. For g, h, i, j, k, l, p, q, and r a two-way RM ANOVA followed by Šídák’s multiple-comparisons test was used; for d, e, f, m, n, and o a REML mixed-effects model followed by Šídák’s multiple-comparisons test; for a-c a one-way analysis of covariance (ANCOVA) was used. Exact *p*-values, sample sizes and statistical details are provided in Supplementary Table 1.

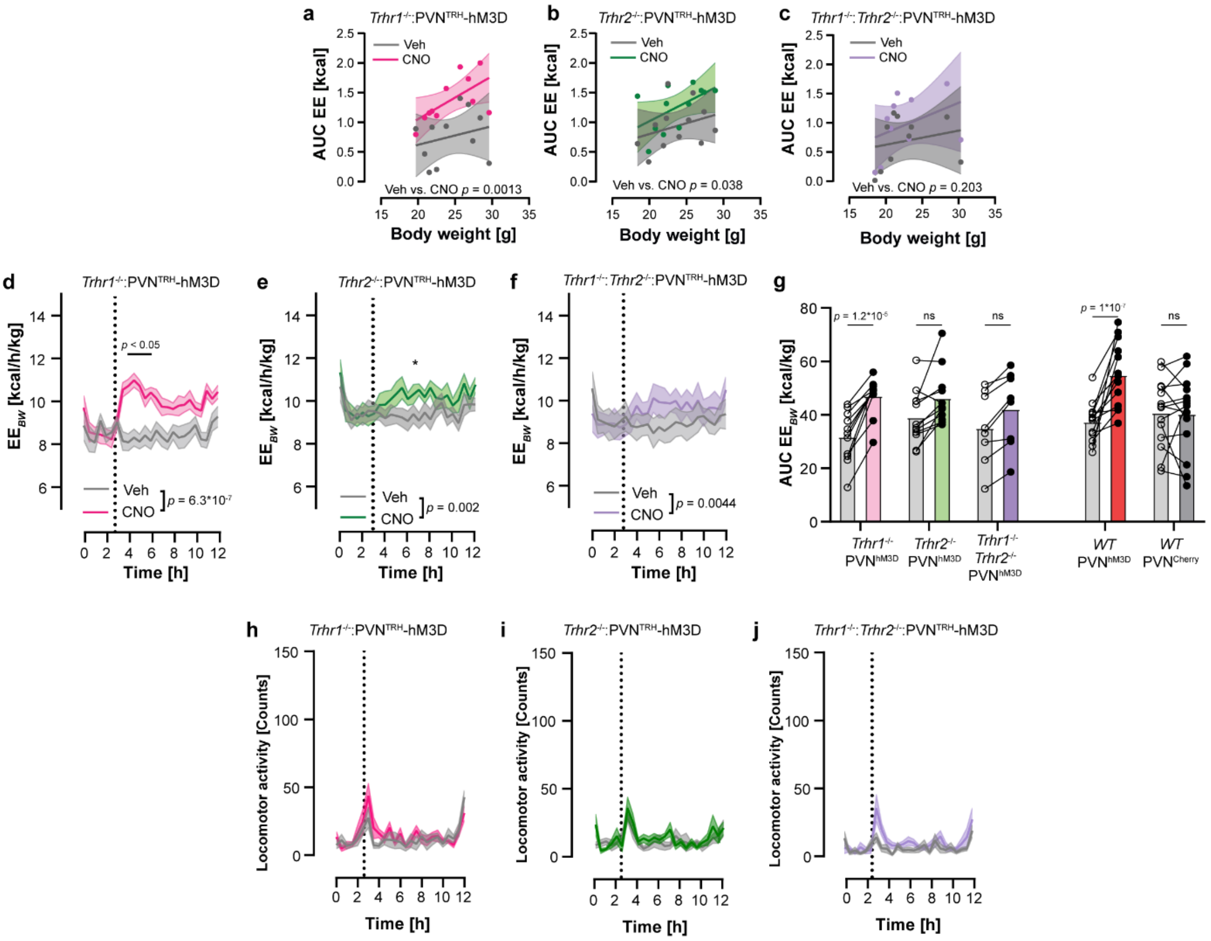


## Supplementary Figure 7

**Importance of TRH-receptors for metabolic effects of PVN$^{TRH}$-hM3D mice. a-c**) Linear regression of the individual changes of the AUC EE from Fig. 7g against the body weight after vehicle (gray) and CNO treatment in *Trhr1*$^{-/-}$:PVN$^{TRH}$-hM3D (**a**, pink), *Trhr2*$^{-/-}$:PVN$^{TRH}$-hM3D (**b**, green) and *Trhr1*$^{-/-}$:*Trhr2*$^{-/-}$:PVN$^{TRH}$-hM3D (**c**, purple) mice for ANCOVA analysis. **d-f**) Changes in energy expenditure normalized by body weight (EE$_{BW}$) after vehicle (0.9 % NaCl, gray) or CNO treatment (3 mg/kg$_{bw}$, i.p., colored lines) over time in *Trhr1*$^{-/-}$:PVN$^{TRH}$-hM3D (**d**, pink), *Trhr2*$^{-/-}$:PVN$^{TRH}$-hM3D (**e**, green) and *Trhr1*$^{-/-}$:*Trhr2*$^{-/-}$:PVN$^{TRH}$-hM3D (**f**, purple) mice. The dotted line indicates injection time (i.p.) in a crossover design on two consecutive days. **g**) Individual changes in EE$_{BW}$ between vehicle and CNO treatment are shown as AUC of EE$_{BW}$ curves from d-f. Changes in home cage locomotor activity after CNO and vehicle treatment over time in *Trhr1*$^{-/-}$:PVN$^{TRH}$-hM3D (**h**, pink), *Trhr2*$^{-/-}$:PVN$^{TRH}$-hM3D (**i,** green) and *Trhr1*$^{-/-}$:*Trhr2*$^{-/-}$:PVN$^{TRH}$-hM3D (**j**, purple) mice. Individual vehicle treatment with NaCl as control (gray). All data presented shown as mean ± s.e.m. of the biological replicates for each group. For d, e, f, g, h, i, and j a two-way RM ANOVA followed by Šídák's multiple-comparisons test, for a-c a one-way analysis of covariance (ANCOVA) was used. Exact *p*-values, sample sizes and statistical details are provided in Supplementary Table 1.

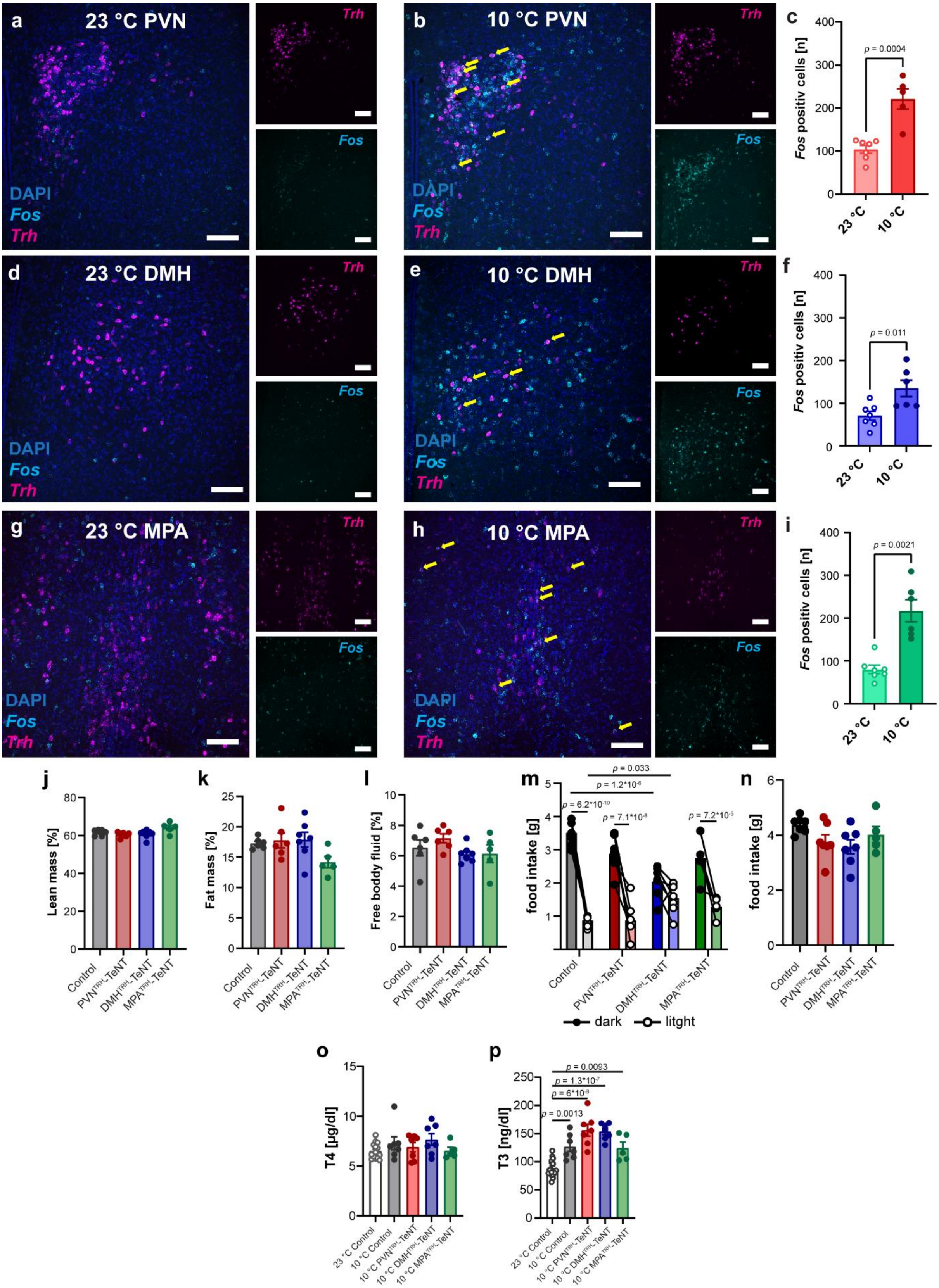
a
23 °C PVN
b
10 °C PVN
c
d
23 °C DMH
e
10 °C DMH
f
g
23 °C MPA
h
10 °C MPA
i
DAPI
Fos
Trh
Fos positiv cells [n]
p = 0.0004
p = 0.011
p = 0.0021
23 °C
10 °C
j
k
l
m
n
o
p
Lean mass [%]
Fat mass [%]
Free boddy fluid [%]
food intake [g]
Control
PVNTRH-TeNT
DMHTRH-TeNT
MPATRH-TeNT
p = 0.033
p = 1.2*10-6
p = 6.2*10-10
p = 7.1*10-8
p = 7.2*10-5
dark
litght
T4 [µg/dl]
T3 [ng/dl]
23 °C Control
10 °C Control
10 °C PVNTRH-TeNT
10 °C DMHTRH-TeNT
10 °C MPATRH-TeNT
p = 0.0093
p = 1.3*10-7
p = 6*10-8
p = 0.0013

## Supplementary Figure 8

**Validation of the cold tolerance test in TeNT mice. a-i**) Expression of *Fos* (cyan) and *Trh* (magenta) mRNA in the PVN (**a-c,** n = 5-7), DMH (**d-f,** n = 6-7) and MPA (**g-l,** n = 6-7) at 23 °C (**a, d**, and **g**) or an ambient temperature of 10 °C for 4 hours (**b**, **e**, and **h**). Number of *Fos* positive cells were quantified for PVN (**c**), DMH (**f**), and MPA (**i**). *Fos* and *Trh* colocalizations are indicated by arrows. Nuclei were visualized by DAPI (blue). Scale bar: 100 µm. **j-l**) Changes in lean (**j**) and fat (**k**) mass and body fluid (**l**) of control, $PVN^{TRH}$-TeNT, $DMH^{TRH}$-TeNT, and $MPA^{TRH}$-TeNT mice 15 days after AAV injection. **m**) Animal individual food intake in the dark and light phase of control, $PVN^{TRH}$-TeNT, $DMH^{TRH}$-TeNT, and $MPA^{TRH}$-TeNT mice as a mean of 72 hours starting 12 days after AAV injection. **n**) Food intake over 24 hours. **o-p**) T4 (**o**) and T3 (**p**) plasma levels in control, $PVN^{TRH}$-TeNT, $DMH^{TRH}$-TeNT, and $MPA^{TRH}$-TeNT mice after 4 hours at 10 °C compared with control animals housed at 23 °C. All data presented shown as mean ± s.e.m. of the biological replicates for each group. For c, e, and i a two-tailed paired t test; for j, k, l, m, n, and o a one-way ANOVA followed by Šídák's (j and m) or Tukey´s (p) multiple comparisons test was used. Exact *p*-values, sample sizes and statistical details are provided in Supplementary Table 1.

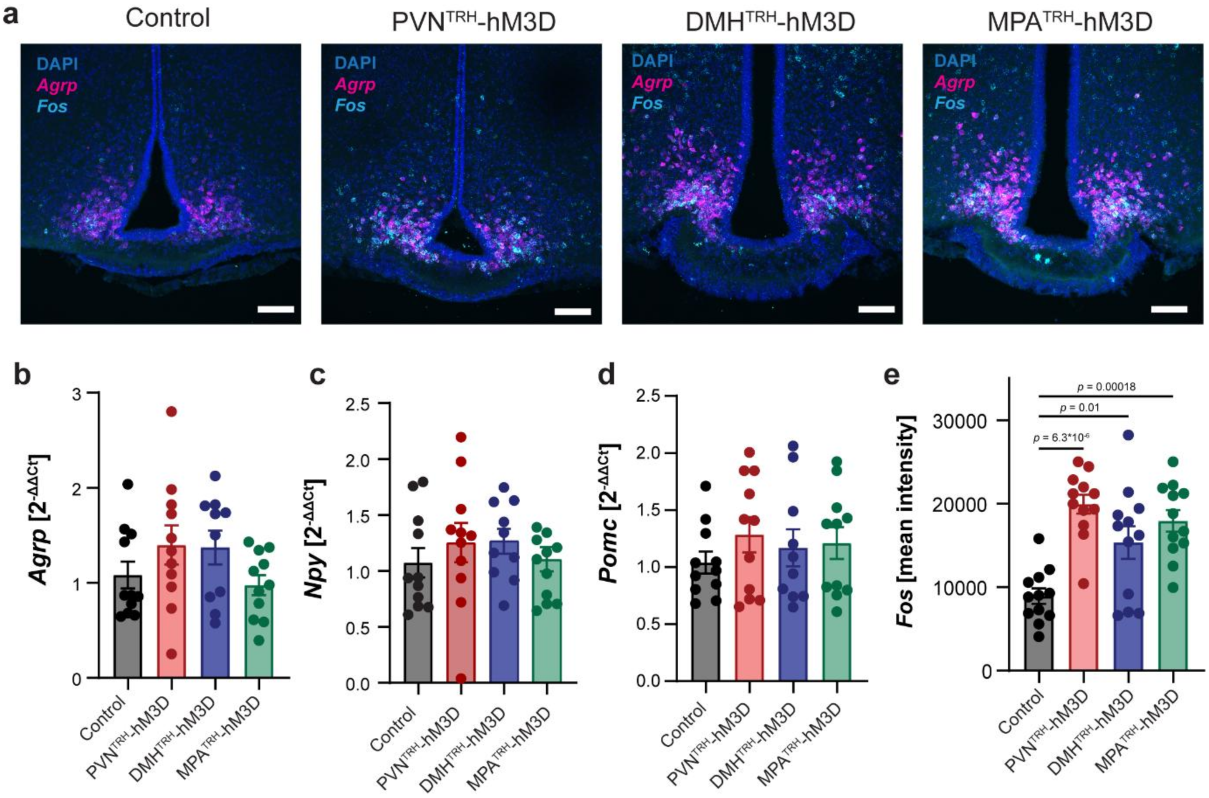


## Supplementary Figure 9

**Effects of activated TRH-neurons on the nucleus arcuate. a**) Exemplary RNAScope for *Fos* (cyan) and *Agrp* (magenta) mRNA of control, PVN$^{TRH}$-hM3D, DMH$^{TRH}$-hM3D, and MPA$^{TRH}$-hM3D mice 3 hours after CNO treatment. Nuclei were visualized by DAPI (blue). Scale bar: 100µm. **b-d**) Expression of *Agrp* (**a**), *Npy* (**c**) and *Pomc* (**d**) of micro dissected arcuate nucleus of control, PVN$^{TRH}$-hM3D, DMH$^{TRH}$-hM3D, and MPA$^{TRH}$-hM3D mice 3 hours after CNO treatment. **e**) Quantification of *Fos* RNAScope in the ARC of control, PVN$^{TRH}$-hM3D, DMH$^{TRH}$-hM3D, and MPA$^{TRH}$-hM3D mice 3 hours after CNO treatment. All data presented are shown as mean ± s.e.m. of the biological replicates for each group. Animal-specific changes under vehicle compared to CNO treatment are indicated by connected data points. For b, c, d, and e a one-way ANOVA followed by Tukey´s (e) multiple comparisons test was used. Exact *p*-values, sample sizes and statistical details are provided in Supplementary Table 1.

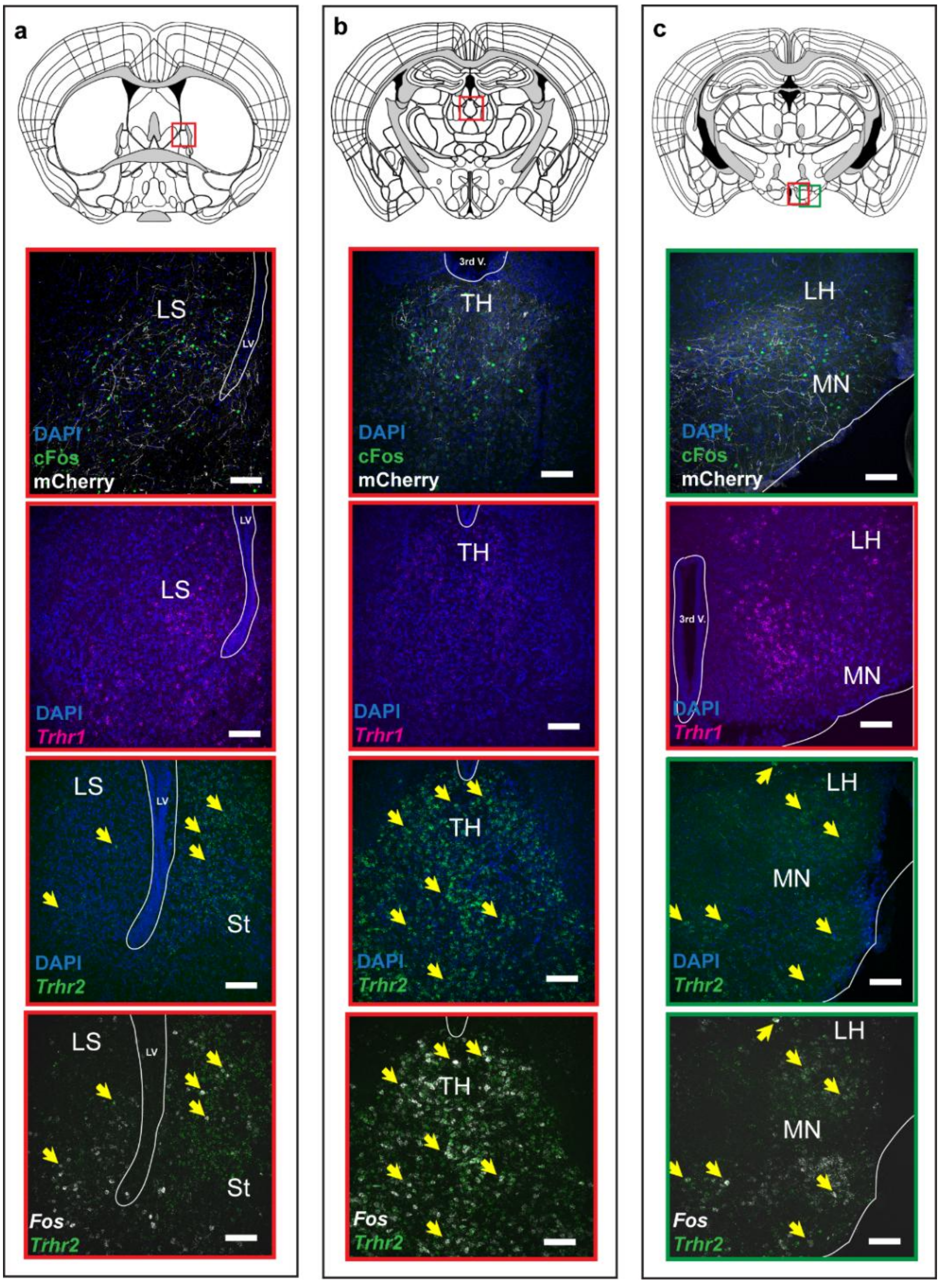


## Supplementary Figure 10

**Visualization of mCherry positive projections** (white) of PVN$^{TRH}$-hM3D mice 180 minutes after CNO injection in combination of immunohistochemistry against cFos (green) in the area of the lateral septum (LS) and striatum (St, **a**), thalamus (TH, **b**) mammillary nucleus (MN) and lateral hypothalamus (LH, **c**). In all three areas, the expression of *Trhr2* can be visualized by RNAScope together with *Fos* mRNA expression. In the thalamus as well as in the lateral area of the mammillary nucleus the *Trhr2* is the dominant TRH-receptor. In the lateral septum the *Trhr1* is dominant. However lateral of the lateral ventricle (LV), in the area of the bed nucleus of the stria terminalis, the *Trhr2* is dominant with less mCherry positive projections from the PVN. Scale bar: 100 µm, nuclei visualized by DAPI (blue). All staining and RNAScops were repeated in 3 independent animals. Drawings in a, b, and c were from Allen Reference Atlas – Mouse Brain [Adult Mouse]. Available from atlas.brain-map.org[1].

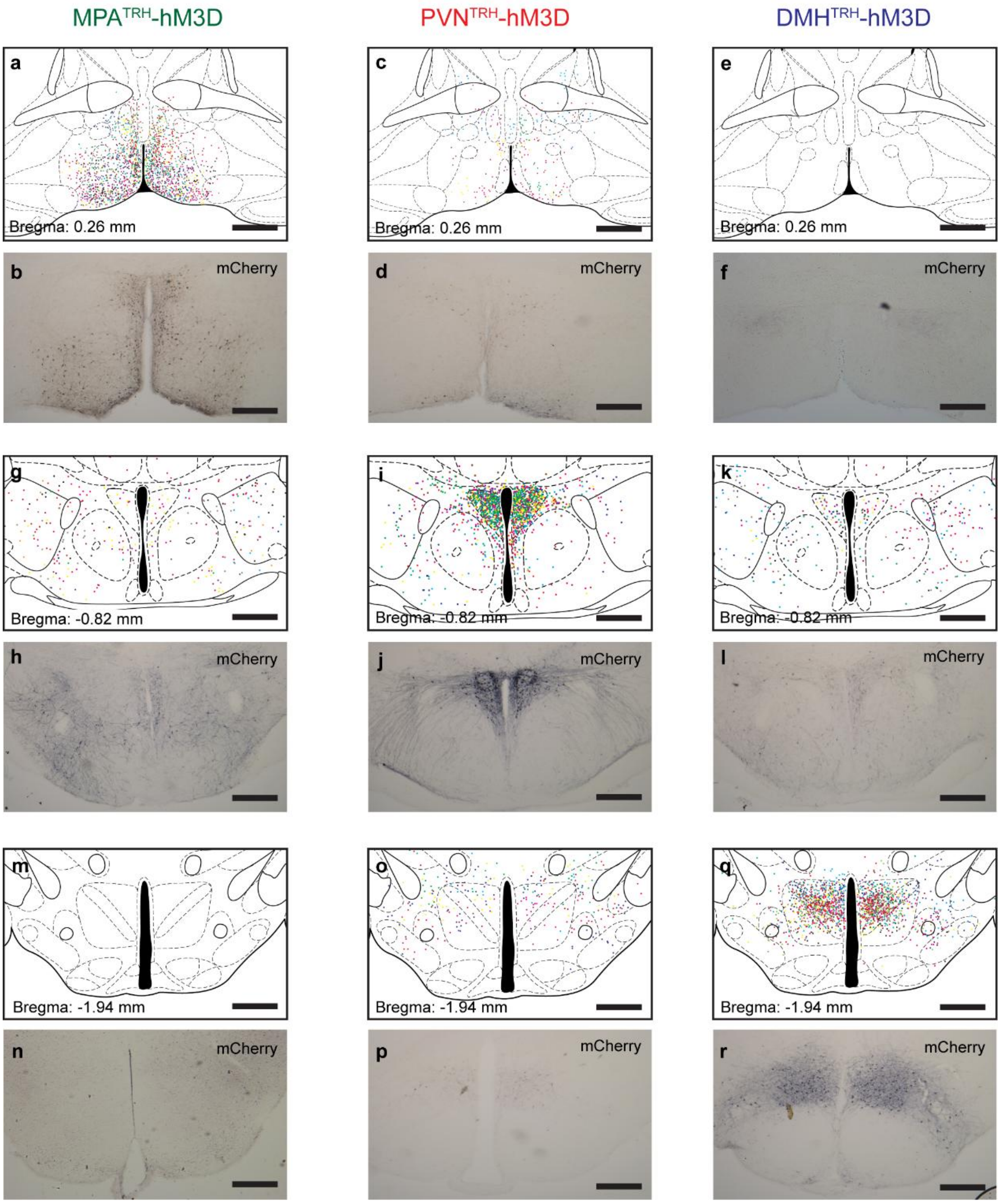


## Supplementary Figure 11

**Validation of the AAV injection sides**. Visualization of the viral spread in the brain of MPA$^{TRH}$-hM3D (**a**, **b, g, h, m,** and **n**), PVN$^{TRH}$-hM3D (**c, d, i, j, o,** and **p**), and DMH$^{TRH}$-hM3D (**e, f, k, l, q,** and **r**) mice in the area of MPA (**a-f**), PVN (**g-l**) and DMH (**m-r**). Each dot in the overview drawings represent an mCherry positive soma as visualized by DAB immunostaining (**a, c, e, g, i, k, m, o,** and **q**). Each color represents one animal (n = 12 animals/group). Microscope pictures are representative images of the regions of interest (**b, d, f, h, j, l, n, p,** and **r**). Scale bar: 500 μm.

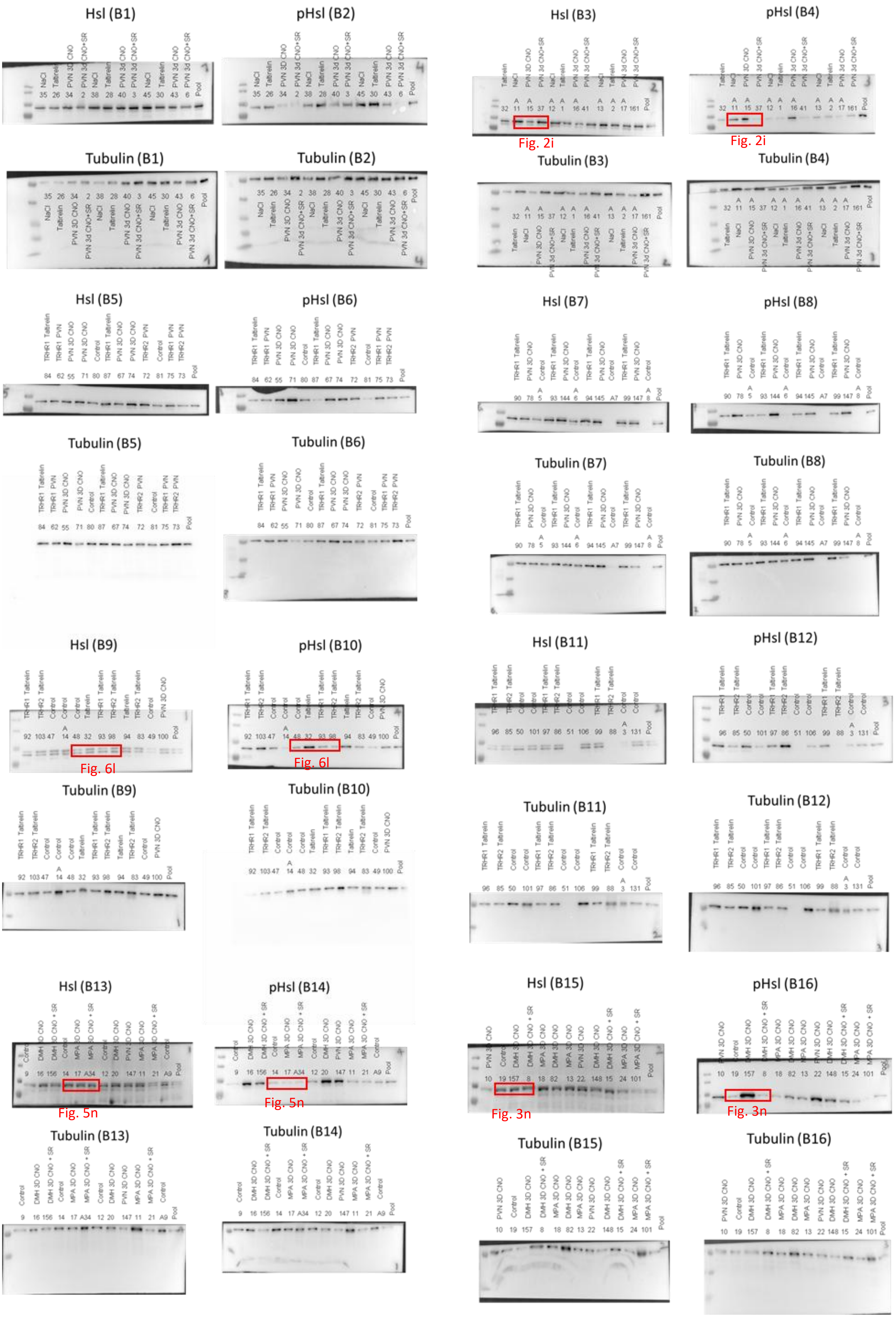
Hsl (B1)
pHsl (B2)
Hsl (B3)
pHsl (B4)
Fig. 2i
Fig. 2i
Tubulin (B1)
Tubulin (B2)
Tubulin (B3)
Tubulin (B4)
Hsl (B5)
pHsl (B6)
Hsl (B7)
pHsl (B8)
Tubulin (B5)
Tubulin (B6)
Tubulin (B7)
Tubulin (B8)
Hsl (B9)
pHsl (B10)
Hsl (B11)
pHsl (B12)
Fig. 6l
Fig. 6l
Tubulin (B9)
Tubulin (B10)
Tubulin (B11)
Tubulin (B12)
Hsl (B13)
pHsl (B14)
Hsl (B15)
pHsl (B16)
Fig. 5n
Fig. 5n
Fig. 3n
Fig. 3n
Tubulin (B13)
Tubulin (B14)
Tubulin (B15)
Tubulin (B16)

## Supplementary Figure 12

### Overview of all Western blot membranes

Images of the analyzed Western blot membranes (B1 to B16) of HSL and pHSL from BAT samples. Each sample was loaded twice to quantify HSL (odd-numbered) and pHSL (even-numbered) on separate membranes. Each loaded sample was additionally analyzed for alpha-tubulin as a loading control. To compare the membranes each membrane was loaded with a pooled internal control (Pool). The squares indicate the protein bands shown in the main images.

a

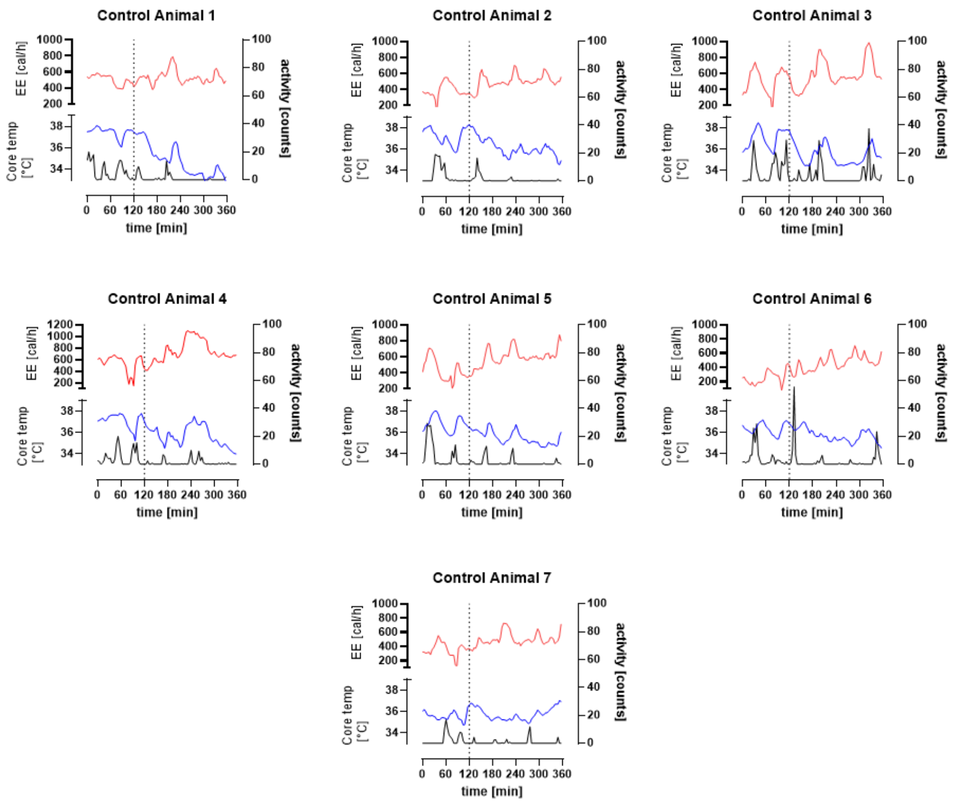


b

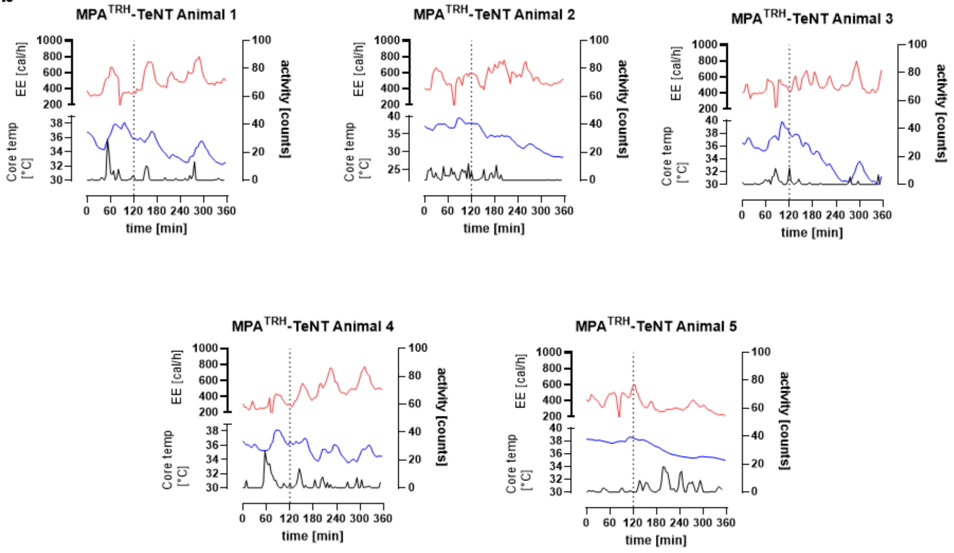


## Supplementary Figure 13 (1/2) (a and b)

Animal individual traces of energy expenditure (EE, red), core body temperature (Core temp, blue), and activity counts (black) during the cold tolerance test in control animals (**a**), MPA$^{TRH}$-TeNT (**b**), PVN$^{TRH}$-TeNT (c), and DMH$^{TRH}$-TeNT (d) mice. The reduction in ambient temperature from 23 °C to 10 °C is marked by the dashed line.

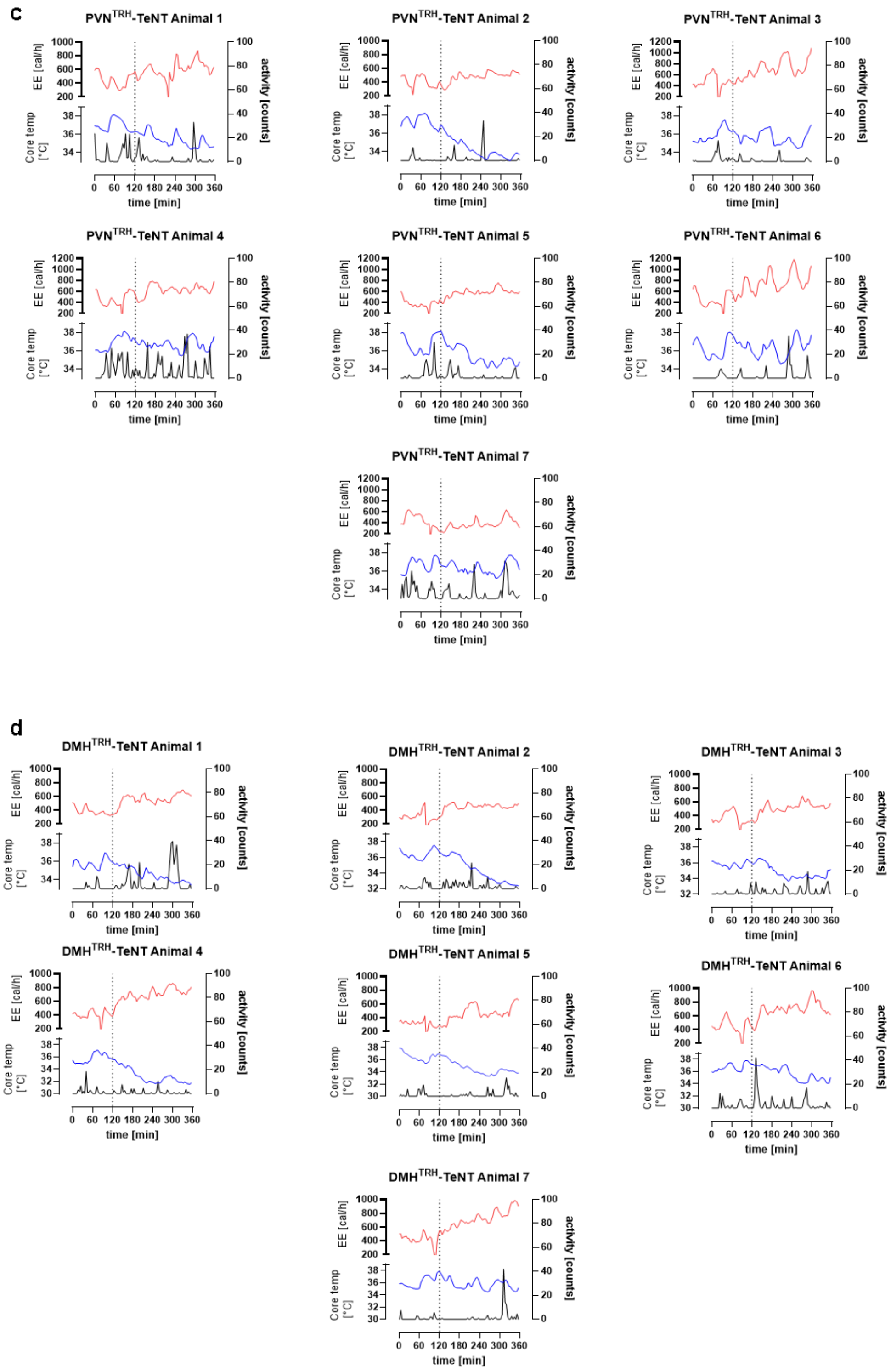


## Supplementary Figure 13 (2/2) (c and d)

Animal individual traces of energy expenditure (EE, red), core body temperature (Core temp, blue), and activity counts (black) during the cold tolerance test in control animals (a), MPA$^{TRH}$-TeNT (b), PVN$^{TRH}$-TeNT (**c**), and DMH$^{TRH}$-TeNT (**d**) mice. The reduction in ambient temperature from 23 °C to 10 °C is marked by the dashed line.

## Supplementary Table 1. Statistical analysis

| Figure | Sample size [n] | Statistical test | Values |
|---|---|---|---|
| 2d | Control: 15 (f:7/m:8)<br>CNO: 16 (f:7/m:9) | Two-tailed unpaired t test (Welch corrected) | *T* (17.5) = 5.308, ***p* = 0.000052** |
| 2e | Control: 16 (f:8/m:8)<br>CNO: 28 (f:14/m:14) | Two-tailed unpaired t test | *T* (42) = 4.883, ***p* = 0.000016** |
| 2f | Control: 16 (f:8/m:8)<br>CNO: 27 (f:14/m:13) | Two-tailed unpaired t test (Welch corrected) | *T* (40.94) = 5.796, ***p* = $5.2*10^{-8}$** |
| 2h | Vehicle: 9 (f:3/m:6)<br>CNO: 9 (f:3/m:6)<br>CNO+SR 59230A: 8 (f:3/m:5) | Two-way RM ANOVA<br><br>followed by<br><br>Šídák's multiple comparisons test | **Genotype**: *F* (2, 22) = 2.004, ***p* = 0.1586**<br>**Treatment**: *F* (1, 22) = 4.810, ***p* = 0.0392**<br>**Genotype x Treatment**: *F* (2, 22) = 3.684, ***p* = 0.0417**<br>Subject: *F* (22, 22) = 1,440, *p* = 0.1996<br><br>Basal vs. NaCl: *p* = 0.7852<br>**Basal vs. CNO**: ***p* = 0.0016**<br>Basal vs. CNO + SR59230A: *p* = 0.9225<br><br>Treated genotypes<br>Control NaCl vs. $PVN^{hM3D}$ CNO: *p* = 0.1865<br>Control NaCl vs. $PVN^{hM3D}$ CNO + SR59230A: *p* = 0.548<br>**$PVN^{hM3D}$ CNO vs. $PVN^{hM3D}$ CNO + SR59230A: *p* = 0.0143** |
| 2i | Vehicle: 14 (f:6/m:8)<br>CNO: 16 (f:7/m:9)<br>CNO+SR 59230A: 6 (f:3/m:3) | Two-sided Brown-Forsythe ANOVA test<br><br>followed by<br><br>Dunnett's T3 multiple comparisons test | *F* (2, 18.80) = 20.30, ***p* = 0.00002**<br><br>**Vehicle vs. CNO: *p* = 0.0009**<br>Vehicle vs. CNO + SR59230A: *p* = 0.8338<br>**CNO vs. CNO + SR59230A: *p* = 0.0005** |
| 2j | 19 (f:10/m:9) | Two-way RM ANOVA with Greenhouse-Geisser correction<br><br>followed by<br><br>Šídák's multiple comparisons test | **Time**: *F* (5.985, 107.7) = 3.712, ***p* = 0.0022**<br>**Treatment**: *F* (1, 18) = 23.62, ***p* = 0.00013**<br>**Time x Treatment**: *F* (6.615, 119.1) = 7.290, ***p* = $5*10^{-7}$**<br><br>Vehicle vs CNO<br>4.0 h: *p* = 0.0085<br>4.5 h: *p* = 0.0093<br>5.5 h: *p* = 0.03<br>6.0 h: *p* = 0.0007<br>6.5 h: *p* = 0.0178<br>7.0 h: *p* = 0.072<br>7.5 h: *p* = 0.0114<br>8.5 h: *p* = 0.0336<br>10.5 h: *p* = 0.0166<br>All other timepoints: *p* > 0.05 |
| 2k | 19 (f:10/m:9) | Two-tailed paired t test | *T* (18) = 5.243, ***p* = $5.5*10^{-5}$** |
| 2l | 19 (f:10/m:9) | One-way analysis of covariance (ANCOVA) | **Vehicle vs CNO after adjustment:**<br><br>*F* (1.0, 35.0) = 17.03, ***p* = 0.00019**<br><br>Covariate (body weight): *F* (1.0, 35.0) = 0.03, *p* = 0.853 |
| 2m | 21 (f:11/m:10) | Mixed-effects model (REML; for missing datapoints)<br><br>with Greenhouse-Geisser correction<br><br>followed by<br><br>Šídák's multiple comparisons test | **Time**: *F* (5.778, 115.6) = 18.15, ***p* = $1.9*10^{-14}$**<br>**Treatment**: *F* (1, 20) = 17.72, ***p* = 0.0004**<br>**Time x Treatment**: *F* (5.01, 93.18) = 2.498, ***p* = 0.036**<br><br>Vehicle vs CNO<br>5.0 h: *p* = 0.0009<br>5.5 h: *p* = 0.0036<br>6.0 h: *p* = 0.0022<br>6.5 h: *p* = 0.0026<br>All other timepoints: *p* > 0.05 |
| 2n | 20 (f:11/m:9) | Two-tailed paired t test | *T* (19) = 2.431, ***p* = 0.0251** |
| 2o | 17 (f:10/m:7) | Two-way RM ANOVA | **Time**: *F* (2.436, 38.97) = 18.15, ***p* = 0.0005**<br>**Treatment**: *F* (1, 16) = 5.716, ***p* = 0.0295** |

| | | | |
|---|---|---|---|
| | | with Greenhouse-Geisser correction<br><br>followed by<br><br>Šídák's multiple comparisons test | Time x Treatment: *F* (2.029, 32.46) = 1.586, *p* = 0.22<br><br>Vehicle vs CNO<br>All timepoints: *p* > 0.05 |
| 2p | 13 (f:7/m:6) | Two-way RM ANOVA<br>with Greenhouse-Geisser correction<br><br>followed by<br><br>Šídák's multiple comparisons test | **Time**: *F* (2.547, 30.56) = 84.92, ***p* = $3.1*10^{-14}$**<br>**Treatment**: *F* (1, 12) = 67.11, ***p* = $2.9*10^{-6}$**<br>**Time x Treatment**: *F* (3.044, 36.53) = 50.65, ***p* = $3.7*10^{-13}$**<br><br>Vehicle vs CNO<br>0.5 h: *p* = 0.0164<br>1.0 h: *p* = 0.0184<br>1.5 h: *p* = 0.0025<br>2.0 h: *p* = 0.0003<br>All other timepoints: *p* < 0.0001 |
| 2q | 13 (f:7/m:6) | Two-tailed paired t test | *T* (12) = 10.61, ***p* = $1.9*10^{-7}$** |
| 2r | 13 (f:7/m:6) | Two-tailed paired t test | *T* (12) = 1.233, *p* = 0.2413 |
| 2s | 20 (f:11/m:9) | Two-way RM ANOVA<br>with Greenhouse-Geisser correction<br><br>followed by<br><br>Šídák's multiple comparisons test | **Time**: *F* (3.861, 73.35) = 11.33, ***p* = $4.6*10^{-7}$**<br>**Treatment**: *F* (1, 19) = 94.47, ***p* = $8.3*10^{-9}$**<br>**Time x Treatment**: *F* (6.290, 119.5) = 34.44, ***p* < $10^{-15}$**<br><br>Vehicle vs CNO<br>5.0 h to 12.0 h: *p* < 0.0001<br>All other timepoints: *p* > 0.05 |

| Figure | Sample size [n] | Statistical test | Values |
|---|---|---|---|
| 3d | Control: 14 (f:8/m:6)<br>CNO: 19 (f:9/m:10) | Two-tailed unpaired t test | *T* (31) = 0.2879, *p* = 0.7754 |
| 3e | Control: 16 (f:8/m:8)<br>CNO: 18 (f:8/m:10) | Two-tailed unpaired t test | *T* (32) = 1.1317, *p* = 0.1971 |
| 3f | Control: 16 (f:8/m:8)<br>CNO: 14 (f:6/m:8) | Two-tailed unpaired t test | *T* (28) = 1.971, *p* = 0.0587 |
| 3g | 12 (f:5/m:7) | Two-way RM ANOVA<br>with Greenhouse-Geisser correction<br><br>followed by<br><br>Šídák's multiple comparisons test | **Time**: *F* (6.391, 70.30) = 1.818, *p* = 0.1035<br>**Treatment:** *F* (1, 11) = 31.34, ***p* = 0.0002**<br>Time x Treatment: *F* (7.227, 79.5) = 2.435, ***p* = 0.0246**<br><br>Vehicle vs CNO<br>5.0 h: *p* = 0.0004<br>6.0 h: *p* = 0.0426<br>9.0 h: *p* = 0.0188<br>All other timepoints: *p* > 0.05 |
| 3h | 12 (f:5/m:7) | Two-tailed paired t test | *T* (11) = 6.932, ***p* = 0.000024** |
| 3i | 12 (f:5/m:7) | One-way analysis of covariance (ANCOVA) | **Vehicle vs CNO after adjustment:**<br><br>*F* (1.0, 21.0) = 34.54, ***p* = $7.9*10^{0}$**<br><br>Covariate (body weight): F (1.0, 21.0) = 4.55, ***p* = 0.045** |
| 3j | 12 (f:5/m:7) | Two-way RM ANOVA<br>with Greenhouse-Geisser correction<br><br>followed by<br><br>Šídák's multiple comparisons test | **Time:** *F* (4.599, 50.59) = 12.42, ***p* = $1,4*10^{-7}$**<br>**Treatment**: *F* (1, 11) = 29.76, ***p* = 0.0002**<br>**Time x Treatment**: *F* (3.614, 39.76) = 2.5, ***p* = 0.0632**<br><br>Vehicle vs CNO<br>4.0 h: *p* = 0.0027<br>4.5 h: *p* = 0.0166<br>5.0 h: *p* = 0.0087 |

| | | | |
|---|---|---|---|
| | | | 6.0 h: *p* = 0.0336<br>All other timepoints: *p* > 0.05 |
| 3k | 12 (f:5/m:7) | Two-tailed paired t test | *T* (11) = 5.372, ***p* = 0.0002** |
| 3m | Vehicle: 9 (f:3/m:6)<br>CNO: 24<br>CNO+SR 59230A: 9 (f:1/m:8) | Two-way RM ANOVA<br><br>followed by<br><br>Šídák's multiple comparisons test | **Genotype**: *F* (2, 39) = 11.04, ***p* = 0.0002**<br>**Treatment**: *F* (1, 39) = 13.56, ***p* = 0.0006**<br>**Genotype** x Treatment: *F* (2, 39) = 3.321, ***p* = 0.0466**<br>**Subject**: *F* (39, 39) = 1.997, ***p* = 0.0181**<br><br>Treatmant:<br>Basal vs. NaCl, *p* = 0.8236<br>**Basal vs. CNO, *p* = 1.4*10$^{-5}$**<br>Basal vs. CNO+SR59230A, *p* = 0.088 |
| 3n | Vehicle: 14 (f:6/m:8)<br>CNO: 5 (f:2/m:3)<br>CNO+SR 59230A: 3 (f:1/m:2) | Two-sided Brown-Forsythe ANOVA test<br><br>followed by<br><br>Dunnett's T3 multiple comparisons test | *F* (2, 4.143) = 14.96, ***p* = 0.0127**<br><br>**Vehicle vs. CNO: *p* = 0.0491**<br>**Vehicle vs. CNO + SR 59230A: *p* = 0.0035**<br>**CNO vs. CNO + SR 59230A: *p* = 0.0324** |
| 3o | 10 (f:4/m:6) | Two-way RM ANOVA with Greenhouse-Geisser correction<br><br>followed by<br><br>Šídák's multiple comparisons test | **Time:** *F* (2.052, 18.47) = 10.94, ***p* = 0.0007**<br>Treatment: *F* (1, 9) = 0.6562, *p* = 0.4388<br>Time x Treatment: *F* (3.022, 27.20) = 0.8817, *p* = 0.4635<br><br>Vehicle vs CNO<br>All timepoints: *p* > 0.05 |
| 3p | 14 (f:6/m:8) | Two-way RM ANOVA with Greenhouse-Geisser correction<br><br>followed by<br><br>Šídák's multiple comparisons test | **Time:** *F* (1.717, 22.32) = 75.59, ***p* = 3.2*10$^{-10}$**<br>**Treatment:** *F* (1, 13) = 81.69, ***p* = 5.7*10$^{-7}$**<br>**Time x Treatment**: *F* (2.553, 33.19) = 73.49, ***p* = 5.1*10$^{-14}$**<br><br>Vehicle vs CNO<br>1.0 h: *p* = 0.0275<br>1.5 h: *p* = 0.0011<br>2.0 h: *p* = 0.0001<br>2.5 h to 6.0 h: *p* < 0.0001<br>All other timepoints: *p* > 0.05 |
| 3q | 14 (f:6/m:8) | Two-tailed paired t test | *T* (13) = 9.994, ***p* = 1.8*10$^{-7}$** |
| 3r | 14 (f:6/m:8) | Two-tailed paired t test | *T* (13) = 0.3587, *p* = 0.7256 |
| 3s | 13 (f:6/m:7) | Two-way RM ANOVA with Greenhouse-Geisser correction<br><br>followed by<br><br>Šídák's multiple comparisons test | **Time**: *F* (4.663, 55.96) = 8.069, ***p* = 1.3*10$^{-5}$**<br>**Treatment:** *F* (1, 12) = 18.63, ***p* = 0.001**<br>**Time x Treatment**: *F* (5.467, 65.60) = 13.65, ***p* = 1.5*10$^{-9}$**<br><br>Vehicle vs CNO<br>6.5 h: *p* = 0.0363<br>7.0 h: *p* = 0.0225<br>7.5 h: *p* = 0.0124<br>8.0 h: *p* = 0.0023<br>8.5 h: *p* = 0.0055<br>9.0 h: *p* = 0.0068<br>9.5 h: *p* = 0.0043<br>10.0 h: *p* = 0.0030<br>10.5 h: *p* = 0.0015<br>11.0 h: *p* = 0.00145<br>All other timepoints: *p* > 0.05 |

| Figure | Sample size [n] | Statistical test | Values |
|---|---|---|---|
| 4c | 8 (f:3/m:5) | Two-way RM ANOVA with Greenhouse-Geisser correction<br><br>followed by<br><br>Šídák's multiple comparisons test | Time: *F* (5.005, 35.04) = 2.306, *p* = 0.0651<br>Treatment: *F* (1, 7) = 1.74, *p* = 0.2286<br>Time x Treatment: *F* (5.126, 35.88) = 1.027, *p* = 0.4174<br><br>Vehicle vs CNO<br>All timepoints: *p* > 0.05 |

| | | | |
|---|---|---|---|
| 4d | 8 (f:3/m:5) | Two-tailed paired t test | *T* (7) = 1.354, *p* =0.2179 |
| 4e | 8 (f:3/m:5) | Mixed-effects model (REML, for missing datapoints)<br>with<br>Greenhouse-Geisser correction<br><br>followed by<br><br>Šídák's multiple comparisons test | Time: *F* (3.451, 24.16) = 1.929, *p* = 0.1452<br>Treatment: *F* (1, 7) = 0.1913, *p* = 0.675<br>Time x Treatment: *F* (3.797, 23.73) = 0.9346, *p* = 0.4571<br><br>Vehicle vs CNO<br>All timepoints: *p* > 0.05 |
| 4f | 7 (f:3/m:4) | Two-tailed paired t test | *T* (12) = 0.3149, *p* = 0.7582 |
| 4g | 8 (f:3/m:5) | Two-way RM ANOVA<br>with<br>Greenhouse-Geisser correction<br><br>followed by<br><br>Šídák's multiple comparisons test | **Time**: *F* (2.056, 14.39) = 16.25, ***p* = 0.0002**<br>Treatment: *F* (1, 7) = 0.936, *p* = 0.359<br>Time x Treatment: *F* (2.185, 15.30) = 0.4030, *p* = 0.6927<br><br>Vehicle vs CNO<br>All timepoints: *p* > 0.05 |
| 4h | 8 (f:3/m:5) | Two-tailed paired t test | *T* (7) = 0.2389, *p* = 0.8180 |
| 4i | 8 (f:3/m:5) | Two-tailed paired t test | *T* (7) = 0.9190, *p* = 0.3887 |
| 4j | 8 (f:3/m:5) | Mixed-effects model (REML, for missing datapoints)<br>with<br>Greenhouse-Geisser correction<br><br>followed by<br><br>Šídák's multiple comparisons test | **Time**: *F* (3.348, 23.43) = 17.51, ***p* = 2*10$^{-6}$**<br>Treatment: *F* (1, 7) = 0.1518, *p* = 0.7084<br>Time x Treatment: *F* (4.478, 29.76) = 1.007, *p* = 0.4254<br><br>Vehicle vs CNO<br>All timepoints: *p* > 0.05 |

| Figure | Sample size [n] | Statistical test | Values |
|---|---|---|---|
| 5d | Control: 15 (f:8/m:7)<br>CNO: 22 (f:9/m:13) | Two-tailed unpaired t test | *T* (35) = 1.598, *p* = 0.1191 |
| 5e | Control: 14 (f:8/m:6)<br>CNO: 22 (f:9/m:13) | Two-tailed unpaired t test | *T* (34) = 1.858, *p* = 0.0719 |
| 5f | Control: 16 (f:9/m:7)<br>CNO: 23 (f:9/m:14) | Two-tailed unpaired t test | *T* (37) = 1.442, *p* = 0.1576 |
| 5g | 15 (f:5/m:10) | Two-way RM ANOVA<br>with Greenhouse-Geisser correction<br><br>followed by<br><br>Šídák's multiple comparisons test | **Time**: *F* (5.363, 75.08) = 14.03, ***p* = 0.0001**<br>**Treatment**: *F* (1, 14) = 25.22, ***p* = 0.0002**<br>**Time x Treatment**: *F* (8.383, 117.4) = 3.421, ***p* = 0.0012**<br><br>Vehicle vs CNO<br>4.0 h: *p* < 0.0001<br>4.5 h: *p* < 0.0001<br>5.0 h: *p* < 0.0001<br>5.5 h: *p* = 0.0002<br>6.0 h: *p* = 0.0011<br>6.5 h: *p* = 0.0042<br>8.0 h: *p* = 0.0308<br>All other timepoints: *p* > 0.05 |
| 5h | 15 (f:5/m:10) | Two-tailed paired t test | *T* (14) = 10.04, ***p* = 8.9*10$^{-8}$** |
| 5i | 15 | One-way analysis of covariance (ANCOVA) | **Vehicle vs CNO after adjustment**:<br><br>*F* (1.0, 27.0) = 22.22, ***p* = 6.3*10$^{-5}$**<br><br>Covariate (body weight): *F* (1.0, 27.0) = 10.44, ***p* = 0.003** |
| 5j | 14 (f:5/m:9) | Two-way RM ANOVA<br>with Greenhouse-Geisser correction<br><br>followed by | **Time**: *F* (4.677, 60.80) = 23.66, ***p* = 9.9*10$^{-13}$**<br>**Treatment**: *F* (1, 13) = 12.28, ***p* = 0.0039**<br>**Time x Treatment**: *F* (5.57, 72.41) = 5.38, ***p* = 0.00018**<br><br>Vehicle vs CNO<br>4.5 h: *p* = 0.0151 |

| | | | |
|---|---|---|---|
| | | Šídák's multiple comparisons test | 5.0 h: $p = 0.0091$<br>5.5 h: $p = 0.026$<br>All other timepoints: $p > 0.05$ |
| 5k | 14 (f:5/m:9) | Two-tailed paired t test | $T$ (13) = 3.586, **$p = 0.0033$** |
| 5m | Vehicle: 9 (f:3/m:6)<br>CNO: 9 (f:5/m:4)<br>CNO+SR 59230A: 9 (f:3/m:6) | Two-way RM ANOVA<br><br>followed by<br><br>Šídák's multiple comparisons test | Genotype: $F$ (2, 24) = 2.401, $p = 0.1121$<br>**Treatment**: $F$ (1, 24) = 7.82, **$p = 0.01$**<br>**Genotype** x **Treatment**: $F$ (2, 24) = 6.8, **$p = 0.046$**<br>**Subject**: $F$ (24, 24) = 2.765, **$p = 0.0181$**<br><br>Treatmant:<br>Basal vs. NaCl, $p = 0.7736$<br>Basal vs. CNO, $p = 0.588$<br>**Basal vs. CNO+SR 59230A, $p = 0.0001$** |
| 5n | Vehicle: 14 (f:6/m:8)<br>CNO: 5 (f:2/m:3)<br>CNO+SR 59230A: 3 (f:1/m:2) | One-way ANOVA test<br><br>followed by<br><br>Tukey's multiple comparisons test | $F$ (2,19) = 1.430, $p = 0.2640$<br><br>Vehicle vs. CNO: $p = 0.3045$<br>Vehicle vs. CNO + SR 59230A: $p = 0.9169$<br>CNO vs. CNO + SR 59230A: $p = 0.2669$ |
| 5o | 16 (f:8/m:8) | Two-way RM ANOVA with Greenhouse-Geisser correction<br><br>followed by<br><br>Šídák's multiple comparisons test | **Time**: $F$ (2.642, 39.64) = 5.486, **$p = 0.0042$**<br>**Treatment**: $F$ (1, 15) = 12.17, **$p = 0.0033$**<br>**Time x Treatment**: $F$ (5.366, 80.49) = 2.828, **$p = 0.0186$**<br><br>Vehicle vs CNO<br>All other timepoints: $p > 0.05$ |
| 5p | 16 (f:8/m:8) | Two-tailed paired t test | $T$ (15) = 3.608, **$p = 0.0026$** |
| 5q | 9 (f:2/m:7) | Two-way RM ANOVA with Greenhouse-Geisser correction<br><br>followed by<br><br>Šídák's multiple comparisons test | **Time**: $F$ (2.087, 16.69) = 58.61, **$p = 2.3*10^{-8}$**<br>**Treatment**: $F$ (1, 8) = 66.27, **$p = 3.9*10^{-5}$**<br>**Time x Treatment**: $F$ (2.839, 22.71) = 44, **$p$ $1.8*10^{-9}$**<br><br>Vehicle vs CNO<br>1.0 h: $p = 0.0169$<br>1.5 h: $p = 0.0171$<br>2.0 h: $p = 0.0071$<br>2.5 h: $p = 0.0070$<br>3.0 h: $p = 0.0009$<br>3.5 h: $p = 0.0001$<br>4.0 h: $p = 0.0004$<br>4.5 h: $p = 0.0005$<br>5.0 h: $p < 0.0001$<br>5.5 h: $p < 0.0001$<br>6.0 h: $p < 0.0001$ |
| 5r | 9 (f:2/m:7) | Two-tailed paired t test | $T$ (8) = 2.871, **$p = 0.0208$** |
| 5s | 9 (f:2/m:7) | Two-tailed paired t test | $T$ (8) = 1.242, $p = 0.2163$ |
| 5t | 15 (f:7/m:8) | Two-way RM ANOVA with Greenhouse-Geisser correction<br><br>followed by<br><br>Šídák's multiple comparisons test | **Time**: $F$ (3.369, 47.17) = 12.39, **$p = 1.9*10^{-6}$**<br>**Treatment**: $F$ (1, 14) = 90.54**, $p = 7*10^{-7}$**<br>**Time x Treatment**: $F$ (3.640, 50.96) = 15.95, **$p = 3.7*10^{-8}$**<br><br>Vehicle vs CNO<br>4.5 h: $p = 0.002$<br>5.0 h to 12.0 h: $p < 0.0001$<br>All other timepoints: $p > 0.05$ |

| Figure | Sample size [n] | Statistical test | Values |
|---|---|---|---|
| 6a | 16 (f:6/m:10) | REML Mixed-effect model (for missing datapoints) with Greenhouse-Geisser correction | **Time**: $F$ (9.810, 147.2) = 15.76, **$p < 10^{-15}$**<br>**Treatment**: $F$ (1, 15) = 15.53, **$p = 0.0013$**<br>**Time x Treatment**: $F$ (8,739, 123.8) = 15.44, **$p < 10^{-15}$** |

| | | | |
|---|---|---|---|
| | | followed by<br>Šídák's multiple comparisons test | NaCl vs Taltirelin<br>4.5 h: $p < 0.0001$<br>5.0 h: $p < 0.0001$<br>5.5 h: $p < 0.0001$<br>6.0 h: $p < 0.0001$<br>6.5 h: $p < 0.0001$<br>7.0 h: $p = 0.0001$<br><br>All other timepoints: $p > 0.05$ |
| 6b | 8 (f:5/m:3) | REML Mixed-effect model (for missing datapoints)<br>with<br>Greenhouse-Geisser correction<br><br>followed by<br>Šídák's multiple comparisons test | **Time**: $F$ (3.799, 26.59) = 2.849, ***p* = 0.0457**<br>Treatment: $F$ (1, 7) = 0.07798, $p$ = 0.7881<br>Time x Treatment: $F$ (4.054, 24.32) = 1.086, $p$ = 0.3860<br><br>NaCl vs Taltirelin<br><br>All timepoints: $p > 0.05$ |
| 6c | 5 (f:2/m:3) | REML Mixed-effect model (for missing datapoints)<br>with<br>Greenhouse-Geisser correction<br><br>followed by<br>Šídák's multiple comparisons test | **Time**: $F$ (3.234, 12.94) = 5.909, ***p* = 0.0082**<br>**Treatment**: $F$ (1, 4) = 2.308, $p$ = 0. 2033<br>**Time x Treatment**: $F$ (3.421, 13.52) = 5.619, ***p* = 0.0086**<br><br>NaCl vs Taltirelin<br>5.5 h: $p$ = 0.0061<br>6.0 h: $p$ = 0.0349<br><br>All other timepoints: $p > 0.05$ |
| 6d | *WT*: 16 (f:6/m:10)<br>*Trhr1*$^{-/-}$: 8 (f:5/m:3)<br>*Trhr2*$^{-/-}$: 5 (f:2/m:3) | Two-way RM ANOVA<br><br>followed by<br><br>Šídák's multiple comparisons test | **Genotype**: $F$ (2, 26) = 44.91, ***p* = 3.7*10$^{-9}$**<br>**Treatment**: $F$ (1, 26) = 84.36, ***p* = 1.2*10$^{-9}$**<br>**Genotype x Treatment**: $F$ (2, 26) = 32.43, ***p* = 8.6*10$^{-8}$**<br>**Subject**: $F$ (26, 26) = 2.881, ***p* = 0.0045**<br><br>NaCl vs Taltirelin<br>***WT*: *p* = 2.2*10$^{-13}$**<br>*Trhr1*$^{-/-}$: $p$ = 0.5362<br>***Trhr2*$^{-/-}$: *p* = 1.6*10$^{-5}$**<br><br>Treatment with Taltirelin<br>***WT* vs. *Trhr1*$^{-/-}$: *p* < 10$^{-15}$**<br>*WT* vs. *Trhr2*$^{-/-}$: ***p* = 0.0018**<br>***Trhr1*$^{-/-}$ vs. *Trhr2*$^{-/-}$: *p* = 5.6*10$^{-7}$**<br><br>All other comparisons: $p > 0.05$ |
| 6e | 16 (f:6/m:10) | Two-way RM ANOVA<br><br>followed by<br><br>Šídák's multiple comparisons test | **Time**: $F$ (5.327, 79.91) = 10.84, ***p* 2.9*10$^{-8}$**<br>**Treatment**: $F$ (1, 15) = 10.71, ***p* = 0.0051**<br>**Time x Treatment**: $F$ (5.284, 79.26) = 18.92, ***p* =4.9*10$^{-5}$**<br><br>NaCl vs Taltirelin<br>6.0 h: $p$ = 0.0281<br>6.5 h: $p$ = 0.0098<br>8.0 h: $p$ = 0.0159<br>8.5 h: $p$ = 0.0264<br><br>All other timepoints: $p > 0.05$ |
| 6f | 8 (f:5/m:3) | REML Mixed-effect model (for missing datapoints)<br>with<br>Greenhouse-Geisser correction<br><br>followed by<br>Šídák's multiple comparisons test | **Time**: $F$ (2.576, 18.03) = 3.970, ***p* = 0.029**<br>Treatment: $F$ (1, 7) = 0.045, $p$ = 0.838<br>Time x Treatment: $F$ (3.389, 22.03) = 0.775, $p$ = 0.5344<br><br>All other timepoints: $p > 0.05$ |
| 6g | 5 (f:2/m:3) | REML Mixed-effect model (for missing datapoints)<br>with<br>Greenhouse-Geisser correction<br><br>followed by<br>Šídák's multiple comparisons test | **Time**: $F$ (3.184, 12.74) = 4.073, ***p* = 0.0295**<br>**Treatment**: $F$ (1, 4) = 27.21, ***p* = 0.0064**<br>**Time x Treatment**: $F$ (2.66, 10.07) = 5.357, ***p* = 0.0203**<br><br>NaCl vs Taltirelin<br>5.5 h: $p$ = 0.0075<br>6.5 h: $p$ = 0.0293 |

| | | | |
|---|---|---|---|
| | | | All other timepoints: *p* > 0.05 |
| 6h | *WT:* 16<br>(f:6/m:10)<br>*Trhr1*$^{-/-}$: 8<br>(f:5/m:3)<br>*Trhr2*$^{-/-}$: 5<br>(f:2/m:3) | Two-way RM ANOVA<br><br>followed by<br><br>Šídák's multiple comparisons test | **Genotype**: *F* (2, 26) = 4.468, ***p* = 0.0215**<br>**Treatment**: *F* (1, 26) = 15.31, ***p* = 0.0006**<br>Genotype x Treatment: *F* (2, 26) = 2.684, *p* = 0.0871<br>Subject: *F* (26, 26) = 1.369, *p* = 0.2141<br><br>NaCl vs Taltirelin<br>***WT: p* = 0.0003**<br>*Trhr1*$^{-/-}$: *p* = 0.6917<br>***Trhr2*$^{-/-}$: *p* = 0.0089**<br><br>Treatment with Taltirelin<br>***WT* vs. *Trhr1*$^{-/-}$: *p* = 0.0022**<br>*WT* vs. *Trhr2*$^{-/-}$: *p* = 0.5804<br>*Trhr1*$^{-/-}$ vs. *Trhr2*$^{-/-}$: *p* = 0.0594<br><br>All other comparisons: *p* > 0.05 |
| 6i | *WT* + NaCl*:* 6<br>(f:3/m:3)<br>*WT* + Taltil: 9<br>(f:3/m:6)<br>*Trhr1*$^{-/-}$: 4<br>(f:0/m:4)<br>*Trhr2*$^{-/-}$: 4<br>(f:0/m:4) | One-way ANOVA<br><br>followed by<br><br>Šídák's multiple comparisons test | *F* (3, 19) = 24.97, ***p* = 8.3*10$^{-7}$**<br><br>***WT* +´NaCl vs. *WT* + Talti: *p* = 7.7*10$^{-6}$**<br>*WT* +´NaCl vs. *Trhr1*$^{-/-}$ + Talti: *p* =0.9948<br>***WT* +´NaCl vs. *Trhr2*$^{-/-}$ + Talti: *p* = 0.001**<br>***WT* +´Talti vs. *Trhr1*$^{-/-}$ + Talti: *p* = 1.3*10$^{-5}$**<br>*WT* +´Talti vs. *Trhr2*$^{-/-}$ + Talti: *p* = 0.8833<br>***Trhr1*$^{-/-}$ + Talti vs. *Trhr2*$^{-/-}$ + Talti: *p* = 0.0008** |
| 6k | *WT* + NaCl: 9<br>(f:3/m:6)<br>*WT:* 8<br>(f:3/m:5)<br>*Trhr1*$^{-/-}$: 7<br>(f:3/m:4)<br>*Trhr2*$^{-/-}$: 6<br>(f:3/m:3) | Two-way RM ANOVA<br><br>followed by<br><br>Šídák's multiple comparisons test | Genotype: *F* (3, 27) = 0.3189, *p* = 0.8116<br>**Treatment**: *F* (1, 27) = 23.57, ***p* = 4.5*10$^{-5}$**<br>**Genotype x Treatment**: *F* (3, 27) = 4.599, ***p* = 0.01**<br>**Subject**: *F* (27, 27) = 4.534, ***p* = 9.6*10$^{-5}$**<br><br>Basal vs Taltirelin<br>***WT: p* = 9.7*10$^{-5}$**<br>*Trhr1*$^{-/-}$: *p* = 0.563<br>***Trhr2*$^{-/-}$: *p* = 0.036**<br>Basal vs NaCl<br>*WT: p* = 0.996 |
| 6l | *WT* + NaCl*:* 12<br>(f:5/m:7)<br>*WT* + Taltil: 6<br>(f:4/m:2)<br>*Trhr1*$^{-/-}$: 12<br>(f:4/m:8)<br>*Trhr2*$^{-/-}$: 6<br>(f:3/m:3) | One-way ANOVA<br><br>followed by<br><br>Šídák's multiple comparisons test | *F* (3, 32) = 9,661, ***p* = 0.0001**<br><br>***WT* +´NaCl vs. *WT* + Talti: *p* = 0.0001**<br>*WT* +´NaCl vs. *Trhr1*$^{-/-}$ + Talti: *p* =0.9994<br>*WT* +´NaCl vs. *Trhr2*$^{-/-}$ + Talti: *p* = 0.4272<br>***WT* +´Talti vs. *Trhr1*$^{-/-}$ + Talti: *p* = 0.0003**<br>***WT* +´Talti vs. *Trhr2*$^{-/-}$ + Talti: *p* = 0.0481**<br>*Trhr1*$^{-/-}$ + Talti vs. *Trhr2*$^{-/-}$ + Talti: *p* = 0.6383 |

| Figure | Sample size [n] | Statistical test | Values |
|---|---|---|---|
| 7a | *Trhr1*$^{-/-}$ Basal: 7<br>*Trhr2*$^{-/-}$ Basal:15<br>*Trhr1*$^{-/-}$:*Trhr2*$^{-/-}$ Basal: 5<br>*Wt* Basal : 15<br><br>*Trhr1*$^{-/-}$ PVN$^{hM3D}$ + CNO: 11<br>(f:4/m:7)<br>*Trhr2*$^{-/-}$ PVN$^{hM3D}$ + CNO: 11<br>(f:6/m:5)<br>*Trhr1*$^{-/-}$::*Trhr2*$^{-/-}$ PVN$^{hM3D}$ +<br>CNO: 10 (f:4/m:6)<br>*WT* PVN$^{hM3D}$ + CNO: 16<br>(f:7/m:9) | Two-way ANOVA<br><br>followed by<br><br>Šídák's multiple comparisons test | Genotype: *F* (3, 82) = 0.1158, *p* = 0.9506<br>**Treatment**: *F* (1, 82) = 14.83, ***p* = 0.0001**<br>**Interaction**: *F* (3, 82) = 7.694, ***p* = 0.0002**<br><br>Basal vs CNO<br>*Trhr1*$^{-/-}$ PVN$^{hM3D}$: *p* = 0.9625<br>***Trhr2*$^{-/-}$ PVN$^{hM3D}$: *p* = 0.0001**<br>*Trhr1*$^{-/-}$::*Trhr2*$^{-/-}$ PVN$^{hM3D}$: *p* = 0.9999<br>***WT* PVN$^{hM3D}$: *p* = 3.5*10$^{-7}$** |
| 7b | *Trhr1*$^{-/-}$ Basal: 9<br>*Trhr2*$^{-/-}$ Basal:21<br>*Trhr1*$^{-/-}$:*Trhr2*$^{-/-}$ Basal: 4<br>*Wt* Basal : 16 | Two-way ANOVA<br><br>followed by | **Genotype**: *F* (3, 102) = 58.56, ***p* < 10$^{-15}$**<br>**Treatment**: *F* (1, 102) = 7.517, ***p* = 0.0072**<br>**Interaction**: *F* (3, 102) = 11.91, ***p* =9.5*10$^{-7}$** |

| | | | |
|---|---|---|---|
| | *Trhr1*$^{-/-}$ PVN$^{hM3D}$ + CNO: 12 (f:4/m:8)<br>*Trhr2*$^{-/-}$ PVN$^{hM3D}$ + CNO: 10 (f:5/m:5)<br>*Trhr1*$^{-/-}$::*Trhr2*$^{-/-}$ PVN$^{hM3D}$ + CNO: 10 (f:4/m:6)<br>*WT* PVN$^{hM3D}$ + CNO: 28 (f:14/m:14) | Šídák's multiple comparisons test | Basal vs CNO<br>*Trhr1*$^{-/-}$ PVN$^{hM3D}$: $p$ = 0.5874<br>***Trhr2*$^{-/-}$ PVN$^{hM3D}$: $p$ = 0.00018**<br>*Trhr1*$^{-/-}$::*Trhr2*$^{-/-}$ PVN$^{hM3D}$: $p$ = 0.6435<br>***WT* PVN$^{hM3D}$: $p$ = $5.5*10^{-9}$** |
| 7c | *Trhr1*$^{-/-}$ Basal: 8<br>*Trhr2*$^{-/-}$ Basal:20<br>*Trhr1*$^{-/-}$:*Trhr2*$^{-/-}$ Basal: 5<br>*Wt* Basal : 16<br><br>*Trhr1*$^{-/-}$ PVN$^{hM3D}$ + CNO: 12 (f:4/m:8)<br>*Trhr2*$^{-/-}$ PVN$^{hM3D}$ + CNO: 11 (f:6/m:5)<br>*Trhr1*$^{-/-}$:*Trhr2*$^{-/-}$ PVN$^{hM3D}$ + CNO: 11 (f:5/m:6)<br>*WT* PVN$^{hM3D}$ + CNO: 27 (f:14/m:13) | Two-way ANOVA<br><br>followed by<br><br>Šídák's multiple comparisons test | **Genotype**: $F$ (3, 101) = 26.24, **$p$ = $1.2*10^{-12}$**<br>**Treatment**: $F$ (1, 101) = 22.93, **$p$ = $5.810^{-6}$**<br>**Interaction**: $F$ (3, 101) = 8.742, **$p$ = $3.3*10^{-5}$**<br><br>Basal vs CNO<br>*Trhr1*$^{-/-}$ PVN$^{hM3D}$: $p$ = 0.9526<br>***Trhr2*$^{-/-}$ PVN$^{hM3D}$: $p$ = 0.004**<br>*Trhr1*$^{-/-}$::*Trhr2*$^{-/-}$ PVN$^{hM3D}$: $p$ = 0.5824<br>***WT* PVN$^{hM3D}$: $p$ = $3.1*10^{-11}$** |
| 7d | Vehicle: 12<br>CNO: 12<br>(f:4/m:8) | Two-way RM ANOVA<br><br>with Greenhouse-Geisser correction<br><br>followed by<br><br>Šídák's multiple comparisons test | **Time**: $F$ (6.034, 66,37) = 3.408, **$p$ = 0.0053**<br>**Treatment:** $F$ (1, 11) = 45.75, **$p$ = $3.1*10^{-5}$**<br>**Time x Treatment**: $F$ (5.398, 59.37) = 4.833, **$p$ = 0.0007**<br><br>Vehicle vs CNO<br>3.5 h: $p$ = 0.0111<br>4.0 h: $p$ = 0.0168<br>4.5 h: $p$ < 0.0027<br>5.0 h: $p$ = 0.0018<br>5.5 h: $p$ = 0.0148<br>6.0 h: $p$ = 0.0354<br>7.5 h: $p$ = 0.0059<br>8.5 h: $p$ = 0.0071<br>All other timepoints: $p$ > 0.05 |
| 7e | Vehicle: 12<br>CNO: 12<br>(f:6/m:6) | Two-way RM ANOVA<br><br>with Greenhouse-Geisser correction<br><br>followed by<br><br>Šídák's multiple comparisons test | **Time**: $F$ (6.514, 71.66) = 2.663, **$p$ = 0.0189**<br>**Treatment**: $F$ (1, 11) = 18.34, **$p$ = 0.0013**<br>**Time x Treatment**: $F$ (5.54, 60.94) = 1.483, $p$ = 0.2032<br><br>Vehicle vs CNO<br>7.0 h: $p$ = 0.0356<br>All other timepoints: $p$ > 0.05 |
| 7f | Vehicle: 10<br>CNO: 10<br>(f:4/m:6) | Two-way RM ANOVA<br><br>with Greenhouse-Geisser correction<br><br>followed by<br><br>Šídák's multiple comparisons test | Time: $F$ (2.593, 23.34) = 1.633, $p$ = 0.2125<br>**Treatment**: $F$ (1, 9) = 13.77, **$p$ = 0.0048**<br>**Time x Treatment**: $F$ (2.285, 20.56) = 1.397, $p$ = 0.2713<br><br>Vehicle vs CNO<br>All timepoints: $p$ > 0.05 |
| 7g | *Trhr1*$^{-/-}$ PVN$^{hM3D}$: 11<br>*Trhr2*$^{-/-}$ PVN$^{hM3D}$: 12<br>*Trhr1*$^{-/-}$:*Trhr2*$^{-/-}$ PVN$^{hM3D}$: 10<br>*WT* PVN$^{hM3D}$: 14<br>*WT* PVN$^{cherry}$: 10 | Two-way RM ANOVA<br><br>followed by<br><br>Šídák's multiple comparisons test | **Genotype:** $F$ (4, 52) = 7.281, **$p$ = $9.8*10^{-5}$**<br>**Treatment**: $F$ (1, 52) = 69.64, **$p$ = $3.6*10^{-11}$**<br>**Genotype x Treatment**: $F$ (4, 52) = 6.528, **$p$ = $2.5*10^{-4}$**<br>**Subject**: $F$ (52, 52) = 2.947, **$p$ = $7.5*10^{-5}$**<br><br>Vehicle vs CNO<br><br>***Trhr1*$^{-/-}$ PVN$^{hM3D}$: $p$ = 0.0004**<br>*Trhr2*$^{-/-}$ PVN$^{hM3D}$: $p$ = 0.0575<br>*Trhr1*$^{-/-}$::*Trhr2*$^{-/-}$ PVN$^{hM3D}$: $p$ = 0.1886<br>***WT PVN*$^{hM3D}$: $p$ = $7.8*10^{-11}$**<br>*WT* PVN$^{cherry}$: $p$ > 0.9999 |
| 7h | Vehicle: 11<br>CNO: 11<br>(f:4/m:7) | Two-way RM ANOVA | **Time**: $F$ (2.686, 26.86) = 9.138, **$p$ = 0.0004**<br>**Treatment**: $F$ (1, 10) = 33.03, **$p$ = 0.0002**<br>Time x Treatment: $F$ (2.4, 24) = 2.253, $p$ = 0.8167 |

| | | | |
|---|---|---|---|
| | | with Greenhouse-Geisser correction<br><br>followed by<br><br>Šídák's multiple comparisons test | Vehicle vs CNO<br>3.5 h: $p$ = 0.0348<br>4.0 h: $p$ = 0.0002<br>4.5 h: $p$ = 0.0008<br>5.0 h: $p$ = 0.034<br>All other timepoints: $p$ > 0.05 |
| 7i | Vehicle: 12<br>CNO: 12<br>(f:6/m:6) | Mixed-effects model (REML, for missing datapoints)<br>with Greenhouse-Geisser correction<br><br>followed by<br><br>Šídák's multiple comparisons test | **Time**: $F$ (4.368, 48.05) = 6.882, **$p$ = 0.0001**<br>**Treatment**: $F$ (1, 11) = 9.753, **$p$ = 0.0097**<br>**Time x Treatment**: $F$ (4.327, 46.16) = 3.243, **$p$ = 0.0175**<br><br>Vehicle vs CNO<br>4.0 h: $p$ = 0.0062<br>4.5 h: $p$ = 0.0096<br>All other timepoints: $p$ > 0.05 |
| 7j | Vehicle: 9<br>CNO: 9<br>(f:4/m:5) | Two-way RM ANOVA<br><br>with Greenhouse-Geisser correction<br><br>followed by<br><br>Šídák's multiple comparisons test | **Time**: $F$ (3.302, 26.41) = 16.99, **$p$ =1.4*10$^{-6}$**<br>**Treatment:** $F$ (1, 8) = 10.48, **$p$ = 0.0119**<br>**Time x Treatment**: $F$ (3.296, 26.37) = 6.155, **$p$ = 0.002**<br><br>Vehicle vs CNO<br>5.0 h: $p$ = 0.0033<br>5.5 h: $p$ = 0.0235<br>6.0 h: $p$ = 0.023<br>All other timepoints: $p$ > 0.05 |
| 7k | *Trhr1*$^{-/-}$ PVN$^{hM3D}$: 11<br>(f:4/m:7)<br>*Trhr2*$^{-/-}$ PVN$^{hM3D}$: 11<br>(f:5/m:6)<br>*Trhr1*$^{-/-}$:*Trhr2*$^{-/-}$ PVN$^{hM3D}$: 9<br>(f:4/m:5)<br>*WT* PVN$^{hM3D}$: 12<br>(f:6/m:6)<br>*WT PVN*$^{cherry}$: 12<br>(f:6/m:6) | Two-way RM ANOVA<br><br>followed by<br><br>Šídák's multiple comparisons test | **Genotype**: $F$ (4, 51) = 8.352, **$p$ = 3.1*10$^{-5}$**<br>**Treatment**: $F$ (1, 51) = 92.62, **$p$ = 5.8*10$^{-13}$**<br>**Genotype x Treatment**: $F$ (4, 51) = 6.064, **$p$ = 0.0005**<br>**Subject**: $F$ (50, 50) = 1.892, **$p$ = 0.013**<br><br>Vehicle vs CNO<br><br>***Trhr1*$^{-/-}$ PVN$^{hM3D}$: $p$ = 1.1*10$^{-8}$**<br>***Trhr2*$^{-/-}$ PVN$^{hM3D}$: $p$ = 0.00033**<br>***Trhr1*$^{-/-}$::*Trhr2*$^{-/-}$ PVN$^{hM3D}$: $p$ = 0.0006**<br>***WT* PVN$^{hM3D}$: $p$ = 0.00099**<br>*WT* PVN$^{cherry}$: $p$ = 0.958 |
| 7o | *Trhr1*$^{-/-}$ PVN$^{hM3D}$: 12<br>(f:4/m:8)<br>*Trhr2*$^{-/-}$ PVN$^{hM3D}$: 11<br>(f:5/m:6)<br>*Trhr1*$^{-/-}$:*Trhr2*$^{-/-}$ PVN$^{hM3D}$: 9<br>(f:4/m:5)<br>*WT* PVN$^{hM3D}$: 11<br>(f:6/m:5)<br>*WT PVN*$^{cherry}$: 14<br>(f:7/m:7) | Two-way RM ANOVA<br><br>followed by<br><br>Šídák's multiple comparisons test | **Genotype**: $F$ (4, 58) = 3.697, **$p$ = 0.0095**<br>**Treatment:** $F$ (1, 58) = 75.38, **$p$ = 4.5*10$^{-12}$**<br>**Genotype x Treatment**: $F$ (4, 58) = 4.074, **$p$ = 0.0056**<br>**Subject**: $F$ (58, 58) = 3.201, **$p$ = 8.7*10$^{-6}$**<br><br>Vehicle vs CNO<br><br>***Trhr1*$^{-/-}$ PVN$^{hM3D}$: $p$ = 0.0053**<br>***Trhr2*$^{-/-}$ PVN$^{hM3D}$: $p$ = 0.0025**<br>***Trhr1*$^{-/-}$::*Trhr2*$^{-/-}$ PVN$^{hM3D}$: $p$ = 0.0066**<br>***WT* PVN$^{hM3D}$: $p$ = 6*10$^{-10}$**<br>*WT* PVN$^{cherry}$: $p$ = 0.3574 |
| 7p | Vehicle: 9<br>CNO: 9<br>(f:4/m:5) | Two-way RM ANOVA<br><br>with Greenhouse-Geisser correction<br><br>followed by<br><br>Šídák's multiple comparisons test | **Time**: $F$ (1.871, 14.97) = 61.80, **$p$ = 7.7*10$^{-8}$**<br>**Treatment:** $F$ (1, 8) = 252.6, **$p$ = 2.5*10$^{-7}$**<br>**Time x Treatment**: $F$ (2.712, 21.69) = 75.62, **$p$ = 2.3*10$^{-11}$**<br><br>Vehicle vs CNO<br>All timepoints: $p$ < 0.0001 |
| 7q | Vehicle: 11<br>CNO: 11<br>(f:6/m:5) | Two-way RM ANOVA<br><br>with Greenhouse-Geisser correction<br><br>followed by<br><br>Šídák's multiple comparisons test | **Time**: $F$ (2.365, 23.65) = 52.74, **$p$ = 6.2*10$^{-10}$**<br>**Treatment**: $F$ (1, 10) = 29.04, **$p$ = 0.0003**<br>**Time x Treatment**: $F$ (2.34, 23.4) = 12, **$p$= 0.0001**<br><br>Vehicle vs CNO<br>1.0 h: p = 0.0478<br>1.5 h: p = 0.0037<br>2.0 h: p = 0.0021<br>3.5 h: p = 0.0322<br>4.0 h: p = 0.0248 |

| | | | |
|---|---|---|---|
| | | | 4.5 h: p = 0.0148<br>5.0 h: p = 0.005<br>5.5 h: p = 0.0016<br>6.0 h: p = 0.0001<br>All timepoints: *p* > 0.05 |
| 7r | Vehicle: 5<br>CNO: 5<br>(f:3/m:2) | Two-way RM ANOVA<br><br>with Greenhouse-Geisser correction<br><br>followed by<br><br>Šídák's multiple comparisons test | **Time**: *F* (1.701, 6.806) = 17.6, ***p* = 0.0024**<br>**Treatment:** *F* (1, 4) = 20.08, ***p* = 0.011**<br>**Time x Treatment**: *F* (2.561, 10.25) = 18.96, ***p* = 0.0002**<br><br>Vehicle vs CNO<br>5.0 h: p = 0.0161<br>5.5 h: p = 0.049<br>6.0 h: p = 0.0348<br>All timepoints: *p* > 0.05 |
| 7s | *Trhr1*$^{-/-}$ PVN$^{hM3D}$: 10<br>(f:4/m:6)<br>*Trhr2*$^{-/-}$ PVN$^{hM3D}$: 10<br>(f:5/m:5)<br>*Trhr1*$^{-/-}$:*Trhr2*$^{-/-}$ PVN$^{hM3D}$: 5<br>(f:3/m:2)<br>*WT* PVN$^{hM3D}$: 13<br>(f:7/m:6)<br>*WT PVN*$^{cherry}$: 15<br>(f:7/m:8) | Two-way RM ANOVA<br><br>followed by<br><br>Šídák's multiple comparisons test | **Genotype**: *F* (4. 47) = 6.022, ***p* = 0.0005**<br>**Treatment**: *F* (1, 47) = 224.2, ***p* < 10$^{-15}$**<br>**Genotype x Treatment**: *F* (4, 47) = 15.29, ***p* = 4.4*10$^{-8}$**<br>**Subject**: F (47, 47) = 2.492, **p = 0.001**<br><br>Vehicle vs CNO<br><br>***Trhr1*$^{-/-}$ PVN$^{hM3D}$: *p* = 3.6*10$^{-13}$**<br>***Trhr2*$^{-/-}$ PVN$^{hM3D}$: *p* = 3*10$^{-7}$**<br>***Trhr1*$^{-/-}$::*Trhr2*$^{-/-}$ PVN$^{hM3D}$: *p* = 1.1*10$^{-5}$**<br>***WT* PVN$^{hM3D}$*: p* = 1*10$^{-12}$**<br>*WT* PVN$^{cherry}$: *p* = 0.3053 |

| Figure | Sample size [n] | Statistical test | Values |
|---|---|---|---|
| 8c | Control: 7<br>(f:3/m:4)<br>PVN$^{TRH}$-TeNT: 7<br>(f:3/m:4)<br>DMH$^{TRH}$-TeNT: 7<br>(f:2/m:5)<br>MPA$^{TRH}$-TeNT: 5<br>(f:2/m:3) | Two-way RM ANOVA<br><br>followed by<br><br>Šídák's multiple comparisons test | **Time**: *F* (15, 330) = 7.963, ***p* = 2*10$^{-15}$**<br>**TRH-Population:** *F* (3, 22) = 16.67, ***p* = 7.1*10$^{-6}$**<br>**Time x TRH-Population**: *F* (45, 330) = 7.666, ***p* < 10$^{-15}$**<br>**Subject:** *F* (22, 330) = 18.91, ***p* < 10$^{-15}$**<br><br>Compared column mean:<br>**Control vs. PVN$^{TRH}$-TeNT**: ***p* = 0.0165**<br>Control vs. DMH$^{TRH}$-TeNT: *p* = 0.9023<br>**Control vs. MPA$^{TRH}$-TeNT**: ***p* = 0.0011**<br><br>Within each row, compare columns:<br><br>Control vs. PVN$^{TRH}$-TeNT:<br>day 7: *p* = 0.019<br>day 8: *p* = 0.0176<br>day 9: *p* = 0.0113<br>day 10: *p* = 0.0152<br>day 11: *p* = 0.0025<br>day 12: *p* = 0.0015<br>day 13: *p* = 0.0463<br>day 14: *p* = 0.0289<br>day 15: *p* = 0.0164<br>All other timepoints: *p* > 0.05<br><br><br>Control vs. DMH$^{TRH}$-TeNT:<br>All timepoints: *p* > 0.05<br><br>Control vs. MPA$^{TRH}$-TeNT:<br>day 5: *p* = 0.0329<br>day 6: *p* = 0.0002<br>day 7: *p* = 0.0001<br>day 8 to 15: *p* < 0.0001<br>All other timepoints: *p* > 0.05 |
| 8d | Control: 6<br>(f:3/m:3)<br>PVN$^{TRH}$-TeNT: 6<br>(f:3/m:3)<br>DMH$^{TRH}$-TeNT: 6<br>(f:2/m:3) | One-way ANOVA<br><br>followed by<br><br>Šídák's multiple comparisons test | F (3, 19) = 6.709, ***p* = 0.0028**<br><br>Control vs. PVN$^{TRH}$-TeNT: *p* = 0.9872<br>Control vs. DMH$^{TRH}$-TeNT: *p* = 0.6718<br>Control vs. MPA$^{TRH}$-TeNT: *p* = 0.0138 |

| | | | |
|---|---|---|---|
| | MPA$^{TRH}$-TeNT: 5<br>(f:2/m:3) | | |
| 8e | Control: 7<br>(f:3/m:4)<br>PVN$^{TRH}$-TeNT: 7<br>(f:3/m:4)<br>DMH$^{TRH}$-TeNT: 7<br>(f:2/m:5)<br>MPA$^{TRH}$-TeNT: 5<br>(f:2/m:3) | Two-way RM ANOVA<br><br>followed by<br><br>Šídák's multiple comparisons test | **Time**: $F$ (89, 1958) = 29.65, **$p < 10^{-15}$**<br>**TRH-Population:** $F$ (3, 22) = 6.687, **$p$ = 0.0022**<br>**Time x TRH-Population**: $F$ (267, 1958) = 4.347, **$p < 10^{-15}$**<br>**Subject**: $F$ (22, 1958) = 45.53, **$p < 10^{-15}$**<br><br>Compare column means:<br><br>Control vs. PVN$^{TRH}$-TeNT: $p$ = 0.999<br>Control vs. DMH$^{TRH}$-TeNT: $p$ = 0.374<br>**Control vs. MPA$^{TRH}$-TeNT: $p$ = 0.0055**<br>PVN$^{TRH}$-TeNT vs. DMH$^{TRH}$-TeNT: $p$ = 0.2992<br>**PVN$^{TRH}$-TeNT vs. MPA$^{TRH}$-TeNT: $p$ = 0.0041**<br>DMH$^{TRH}$-TeNT vs. MPA$^{TRH}$-TeNT: $p$ = 0.2447 |
| 8f | Control: 7<br>(f:3/m:4)<br>PVN$^{TRH}$-TeNT: 7<br>(f:3/m:4)<br>DMH$^{TRH}$-TeNT: 7<br>(f:2/m:5)<br>MPA$^{TRH}$-TeNT: 5<br>(f:2/m:3) | Two-way RM ANOVA<br><br>followed by<br><br>Šídák's multiple comparisons test | **Temperature**: $F$ (1, 22) = 36.40, **$p = 4.5*10^{-6}$**<br>**TRH-Population:** $F$ (3, 22) = 8.778, **$p$ = 0.0005**<br>**Temperature x TRH-Population**:<br>$F$ (3, 22) = 5.871, **$p$ = 0.0042**<br>**Subject**: $F$ (22, 22) = 0.8908, $p$ = 0.6057<br><br>23°C vs. 10°C:<br>Control: $p$ = 0.1425<br>PVN$^{TRH}$-TeNT: $p$ = 0.1683<br>**DMH$^{TRH}$-TeNT: $p$ = 0.0211**<br>**MPA$^{TRH}$-TeNT: $p = 3.8*10^{-6}$**<br><br>**10°C:**<br>Control vs. PVN$^{TRH}$-TeNT: $p$ = 0.999<br>Control vs. DMH$^{TRH}$-TeNT: $p$ = 0.7650<br>**Control vs. MPA$^{TRH}$-TeNT: $p = 5.7*10^{-6}$**<br>PVN$^{TRH}$-TeNT vs. DMH$^{TRH}$-TeNT: $p$ = 0.2992<br>**PVN$^{TRH}$-TeNT vs. MPA$^{TRH}$-TeNT: $p = 2.3*10^{-6}$**<br>**DMH$^{TRH}$-TeNT vs. MPA$^{TRH}$-TeNT: $p$ = 0.0003** |
| 8g-i | Control: 7<br>(f:3/m:4)<br>PVN$^{TRH}$-TeNT: 7<br>(f:3/m:4)<br>DMH$^{TRH}$-TeNT: 7<br>(f:2/m:5)<br>MPA$^{TRH}$-TeNT: 5<br>(f:2/m:3) | Mixed-effects model (REML, for missing datapoints) | **Time**: $F$ (89, 1836) = 17.25, **$p < 10^{-15}$**<br>TRH-Population: $F$ (3, 22) = 0.7430, $p$ = 0.5379<br>**Time x TRH-Population**: $F$ (258, 1836) = 1.335, **$p$ = 0.0007** |
| 8j | Control: 7<br>(f:3/m:4)<br>PVN$^{TRH}$-TeNT: 7<br>(f:3/m:4)<br>DMH$^{TRH}$-TeNT: 7<br>(f:2/m:5)<br>MPA$^{TRH}$-TeNT: 5<br>(f:2/m:3) | Two-way RM ANOVA<br><br>followed by<br><br>Šídák's multiple comparisons test | **Temperature**: $F$ (1, 22) = 87.73, **$p = 3.9*10^{-9}$**<br>**TRH-Population:** $F$ (3, 22) = 4.931, **$p$ = 0.0091**<br>Temperature x TRH-Population:<br>$F$ (3, 22) = 2.873, $p$ = 0.0594<br>**Subject**: $F$ (22, 22) = 1.179, $p$ = 0.3512<br><br>23°C vs. 10°C:<br>**Control: $p$ = 0.0001**<br>**PVN$^{TRH}$-TeNT: $p = 6.5*10^{-6}$**<br>**DMH$^{TRH}$-TeNT: $p = 1*10^{-7}$**<br>MPA$^{TRH}$-TeNT: $p$ = 0.0573<br><br>**10°C:**<br>Control vs. PVN$^{TRH}$-TeNT: $p$ = 0.4886<br>Control vs. DMH$^{TRH}$-TeNT: $p$ = 0.4886<br>**Control vs. MPA$^{TRH}$-TeNT: $p$ = 0.0171**<br>PVN$^{TRH}$-TeNT vs. DMH$^{TRH}$-TeNT: $p$ = 0.9953<br>**PVN$^{TRH}$-TeNT vs. MPA$^{TRH}$-TeNT: $p$ = 0.0008**<br>**DMH$^{TRH}$-TeNT vs. MPA$^{TRH}$-TeNT: $p$ = 0.0008** |
| 8k | 7 (f:3/m:4) | One-way analysis of covariance (ANCOVA) | **23°C vs. 10°C after adjustment:**<br><br>$F$ (1.0, 11.0) = 17.12, **$p$ = 0.002**<br><br>Covariate (body weight): $F$ (1.0, 11.0) = 0.10, $p$ = 0.757 |
| 8l | 7 (f:3/m:4) | One-way analysis of covariance (ANCOVA) | **23°C vs. 10°C after adjustment:** |

| | | | |
|---|---|---|---|
| | | | $F$ (1.0, 11.0) = 93.05, $\mathbf{p = 1.2*10^{-6}}$<br><br>Covariate (body weight): $F$ (1.0, 11.0) = 5.13, $p$ = 0.045 |
| 8m | 7 (f:2/m:5) | One-way analysis of covariance (ANCOVA) | **23°C vs. 10°C after adjustment:**<br><br>$F$ (1.0, 11.0) = 34.91, $\mathbf{p = 1*10^{-4}}$<br><br>Covariate (body weight): $F$ (1.0, 11.0) = 6.25, $p$ = 0.030 |
| 8n | 5 (f:2/m:3) | One-way analysis of covariance (ANCOVA) | 23°C vs. 10°C after adjustment:<br><br>$F$ (1.0, 7.0) = 3.91, $p$ = 0.089<br><br>Covariate (body weight): $F$ (1.0, 7.0) = 1.76, $p$ = 0.226 |

| Figure | Sample size [n] | Statistical test | Values |
|---|---|---|---|
| Suppl. 2d | 21 (f:11/m:10) | Two-way RM ANOVA with Greenhouse-Geisser correction<br><br>followed by<br><br>Šídák's multiple comparisons test | **Time**: $F$ (7.351, 147) = 4.055, $\mathbf{p = 0.0003}$<br>**Treatment**: $F$ (1, 20) = 20.31, $\mathbf{p = 0.0002}$<br>**Time x Treatment**: $F$ (6.423, 128.5) = 5.071, $\mathbf{p = 7.1*10^{-5}}$<br><br>Vehicle vs CNO<br>4.0 h: $p$ = 0.0360<br>4.5 h: $p$ = 0.0044<br>5.5 h: $p$ = 0.0296<br>6.0 h: $p$ = 0.0017<br>6.5 h: $p$ = 0.0061<br>All other timepoints: $p$ > 0.05 |
| Suppl. 2e | 21 (f:11/m:10) | Two-tailed paired t test | $T$ (20) = 4.872, $\mathbf{p = 9.2*10^{-5}}$ |
| Suppl. 2f | 13 (f:7/m:6) | Two-way RM ANOVA<br><br>followed by<br><br>Šídák's multiple comparisons test | **Time**: $F$ (12, 144) = 83.000, $\mathbf{p < 10^{-15}}$<br>**Treatment**: $F$ (2, 24) = 68.25, $\mathbf{p = 1.2*10^{-10}}$<br>**Time x Treatment**: $F$ (24, 288) = 46.01, $\mathbf{p < 10^{-15}}$<br><br>Vehicle vs CNO<br>0.5 h: $p$ = 0.0273<br>1 h to 6 h: $p$ < 0.0001<br>All other timepoints: $p$ > 0.05<br><br><br>Vehicle vs CNO + fasting<br>0.5 h: $p$ = 0.0273<br>1.0 h: $p$ = 0.0028<br>1.5 h to 6 h: $p$ < 0.0001<br>All other timepoints: $p$ > 0.05 |
| Suppl. 2g | 13 (f:7/m:6) | Two-way RM ANOVA with Greenhouse-Geisser correction<br><br>followed by<br><br>Šídák's multiple comparisons test | **Time**: $F$ (5.814, 69.77) = 5.234, $\mathbf{p = 0.0002}$<br>**Treatment**: $F$ (1.402, 16.82) = 6.12, $\mathbf{p = 0.0165}$<br>**Time x Treatment**: $F$ (7.589, 91.06) = 3.057, $\mathbf{p = 0.005}$<br><br>Vehicle vs CNO<br>1.0 h: $p$ = 0.0013<br>1.5 h: $p$ < 0.0001<br>2.5 h: $p$ = 0.0065<br>All other timepoints: $p$ > 0.05<br><br><br>Vehicle vs CNO + fasting<br>1.0 h: $p$ = 0.0002<br>1.5 h: $p$ < 0.0001<br>2.5 h: $p$ = 0.0164<br>All other timepoints: $p$ > 0.05 |
| Suppl. 2h | 13 (f:7/m:6) | Mixed-effects model (REML, for missing datapoints)<br><br>with Greenhouse-Geisser correction<br><br>followed by | **Time**: $F$ (4.508, 54.10) = 11.16, $\mathbf{p = 4.6*10^{-7}}$<br>**Treatment**: $F$ (1.395, 16.74) = 0.9563, $p$ = 0.3732<br>**Time x Treatment**: $F$ (5.307, 60.46) = 3.726, $\mathbf{p = 0.0045}$<br><br>Vehicle vs CNO<br>1.0 h: $p$ = 0.0049<br>1.5 h: $p$ = 0.0433 |

| | | | |
|---|---|---|---|
| | | Šídák's multiple comparisons test | 2.0 h: $p = 0.0292$<br>2.5 h: $p = 0.0369$<br>All other timepoints: $p > 0.05$<br><br>Vehicle vs CNO + fasting<br>0.5 h: $p = 0.0049$<br>1.0 h: $p = 0.0022$<br>1.5 h: $p = 0.0305$<br>All other timepoints: $p > 0.05$ |

| Figure | Sample size [n] | Statistical test | Values |
|---|---|---|---|
| Suppl. 3d | 12 (f:5/m:7) | Two-way RM ANOVA with Greenhouse-Geisser correction<br><br>followed by<br><br>Šídák's multiple comparisons test | **Time**: $F$ (6.515, 71.66) = 2.665, $\boldsymbol{p = 0.0188}$<br>**Treatment:** $F$ (1, 11) = 23.86, $\boldsymbol{p = 0.0005}$<br>Time x Treatment: $F$ (6.269, 68.96) = 1.666, $p = 0.1396$<br><br>Vehicle vs CNO<br>5.0 h: $p = 0.0017$<br>6.0 h: $p = 0.0064$<br>6.5 h: $p = 0.0403$<br>All other timepoints: $p > 0.05$ |
| Suppl. 3e | 12 (f:5/m:7) | Two-tailed paired t test | $T$ (11) = 6.196, $\boldsymbol{p = 6.8*10^{-8}}$ |
| Suppl. 3f | 13 (f:6/m:7) | Two-way RM ANOVA<br><br>followed by<br><br>Šídák's multiple comparisons test | **Time**: $F$ (12, 144) = 49.80, $\boldsymbol{p < 10^{-15}}$<br>**Treatment**: $F$ (2, 24) = 29.29, $\boldsymbol{p = 3.6*10^{-7}}$<br>**Time x Treatment**: $F$ (24, 288) = 13.51, $\boldsymbol{p < 10^{-15}}$<br><br>Vehicle vs CNO<br>2.0 h: $p = 0.0033$<br>2.5 h: $p = 0.0002$<br>3.0 h to 6 h: $p < 0.0001$<br>All other timepoints: $p > 0.05$<br><br>Vehicle vs CNO + fasting<br>4.0 h: $p = 0.0002$<br>4.5 h to 6 h: $p < 0.0001$<br>All other timepoints: $p > 0.05$ |
| Suppl. 3g | 13 (f:6/m:7) | Two-way RM ANOVA with Greenhouse-Geisser correction<br><br>followed by<br><br>Šídák's multiple comparisons test | **Time**: $F$ (6.160, 73.92) = 2.134, $p = 0.0578$<br>**Treatment**: $F$ (1.896, 22.76) = 8.248, $\boldsymbol{p = 0.0023}$<br>**Time x Treatment**: $F$ (9.368, 112.4) = 3.024, $\boldsymbol{p = 0.0025}$<br><br>Vehicle vs CNO<br>2.0 h: $p = 0.0410$<br>2.5 h: $p = 0.0118$<br>3.0 h: $p = 0.0358$<br>All other timepoints: $p > 0.05$<br><br>Vehicle vs CNO + fasting<br>1.0 h: $p = 0.0045$<br>1.5 h: $p = 0.0068$<br>All other timepoints: $p > 0.05$ |
| Suppl. 3h | 13 (f:6/m:7) | Two-way RM ANOVA with Greenhouse-Geisser correction<br><br>followed by<br><br>Šídák's multiple comparisons test | **Time**: $F$ (6.134, 73.60) = 14.04, $\boldsymbol{p = 1.1*10^{-10}}$<br>**Treatment**: $F$ (1.807, 21.68) = 7.425, $\boldsymbol{p = 0.0043}$<br>**Time x Treatment**: $F$ (8.699, 104.4) = 2.834, $\boldsymbol{p = 0.0055}$<br><br>Vehicle vs CNO<br>1.0 h: $p = 0.0216$<br>1.5 h: $p = 0.0011$<br>2.0 h: $p = 0.0063$<br>All other timepoints: $p > 0.05$<br><br>Vehicle vs CNO + fasting<br>0.5 h: $p = 0.0023$<br>1.0 h: $p = 0.0076$<br>All other timepoints: $p > 0.05$ |

| | | | |
|---|---|---|---|

| Figure | Sample size [n] | Statistical test | Values |
|---|---|---|---|
| Suppl. 4a | 24 (f:12/m:12) | Two-way RM ANOVA with Greenhouse-Geisser correction<br><br>followed by<br><br>Šídák's multiple comparisons test | Time: *F* (11.72, 269.6) = 23.67, $p < 10^{-15}$<br>Treatment: *F* (1, 23) = 0.4146, *p* = 0.526<br>Time x Treatment: *F* (11.63, 267.4) = 1.198, *p* = 0.2474<br><br>Vehicle vs CNO<br>All other timepoints: *p* > 0.05 |
| Suppl. 4b | 24 (f:12/m:12) | Two-tailed paired t test | *T* (23) = 0.1867, *p* = 0.8535 |
| Suppl. 4c | 20 (f:10/m:10) | Two-way RM ANOVA with Greenhouse-Geisser correction<br><br>followed by<br><br>Šídák's multiple comparisons test | Time: *F* (9.442, 179.4) = 19.53, $p < 10^{-15}$<br>Treatment: *F* (1, 19) = 1.742, *p* = 0.2026<br>Time x Treatment: *F* (10.04, 190.9) = 1.198, *p* = 0.3186<br><br>Vehicle vs CNO<br>All other timepoints: *p* > 0.05 |
| Suppl. 4d | 20 (f:10/m:10) | Two-tailed paired t test | *T* (19) = 1.897, *p* = 0.0732 |
| Suppl. 4e | 7 (f:3/m:4) | Two-way RM ANOVA with Greenhouse-Geisser correction<br><br>followed by<br><br>Šídák's multiple comparisons test | Time: *F* (2.727, 16.36) = 7.035, *p* = 0.0036<br>Treatment: *F* (1, 6) = 0.2383, *p* = 0.6428<br>Time x Treatment: *F* (3.515, 21.09) = 0.9098, *p* = 0.4660<br><br>Vehicle vs CNO<br>All other timepoints: *p* > 0.05 |
| Suppl. 4f | 15 (f:5/m:10) | Two-tailed paired t test | *T* (14) = 1,096, *p* = 0.2915 |
| Suppl. 4g | 20 (f:10/m:10) | Two-way RM ANOVA<br><br>followed by<br><br>Šídák's multiple comparisons test | Time: *F* (12, 228) = 15.7, $p < 10^{-15}$<br>Treatment: *F* (1, 19) = 0.041, *p* = 0.6428<br>Time x Treatment: *F* (12, 228) = 0.7699, *p* = 0.6811<br><br>Vehicle vs CNO<br>All other timepoints: *p* > 0.05 |
| Suppl. 4h | 24 (f:12/m:12) | Two-way RM ANOVA with Greenhouse-Geisser correction<br><br>followed by<br><br>Šídák's multiple comparisons test | Time: *F* (5.231, 120.3) = 64.22, $p < 10^{-15}$<br>Treatment: *F* (1, 23) = 0.244, *p* = 0.626<br>Time x Treatment: *F* (8.933, 205.4) = 1.367, *p* = 0.2056<br><br>Vehicle vs CNO<br>All other timepoints: *p* > 0.05 |

| Figure | Sample size [n] | Statistical test | Values |
|---|---|---|---|
| Suppl. 5d | 15 (f:5/m:10) | Two-way RM ANOVA with Greenhouse-Geisser correction<br><br>followed by<br><br>Šídák's multiple comparisons test | **Time**: *F* (7.574, 106) = 4.580, ***p* = 0.0001**<br>**Treatment**: *F* (1, 14) = 64.34, $\boldsymbol{p = 1.3 * 10^{-6}}$<br>**Time x Treatment**: *F* (7.834, 109.7) = 6.588, $\boldsymbol{p = 6.4 * 10^{-7}}$<br><br>Vehicle vs CNO<br>4.0 h: *p* < 0.0001<br>4.5 h: *p* < 0.0001<br>5.0 h: *p* < 0.0001<br>5.5 h: *p* = 0.0004<br>6.0 h: *p* = 0.0005<br>6.5 h: *p* = 0.003<br>8.0 h: *p* = 0.022<br>All other timepoints: *p* > 0.05 |
| Suppl. 5e | 15 (f:5/m:10) | Two-tailed paired t test | *T* (13) = 6.631, $\boldsymbol{p = 1.6 * 10^{-6}}$ |

| | | | |
|---|---|---|---|
| Suppl. 5f | 13 (f:5/m:8) | Two-way RM ANOVA<br><br>followed by<br><br>Šídák's multiple comparisons test | **Time**: *F* (12, 144) = 124.9, ***p* < $10^{-15}$**<br>**Treatment**: *F* (2, 24) = 84.59, ***p* = $1.4*10^{-11}$**<br>**Time x Treatment**: *F* (24, 288) = 54.51, ***p* < $10^{-15}$**<br><br>Vehicle vs CNO<br>0.5 h: *p* = 0.0308<br>1.0 h to 6 h: *p* < 0.0001<br>All other timepoints: *p* > 0.05<br><br>Vehicle vs CNO + fasting<br>4.0 h: *p* = 0.0057<br>4.5 h: *p* = 0.0008<br>5.0 h to 6 h: *p* < 0.0001<br>All other timepoints: *p* > 0.05 |
| Suppl. 5g | 13 (f:5/m:8) | Two-way RM ANOVA with Greenhouse-Geisser correction<br><br>followed by<br><br>Šídák's multiple comparisons test | **Time**: *F* 6.744, 87.67) = 2.425, ***p* = 0.00271**<br>**Treatment**: *F* (1.603, 20.84) = 9.241, ***p* = 0.0023**<br>**Time x Treatment**: *F* (9.188, 119.5) = 1.985, ***p* = 0.0456**<br><br>Vehicle vs CNO<br>1.5 h: *p* = 0.008<br>2.5 h: *p* = 0.0019<br>All other timepoints: *p* > 0.05<br><br>Vehicle vs CNO + fasting<br>1.0 h: *p* = 0.0071<br>All other timepoints: *p* > 0.05 |
| Suppl. 5h | 13 (f:5/m:8) | Two-way RM ANOVA with Greenhouse-Geisser correction<br><br>followed by<br><br>Šídák's multiple comparisons test | **Time**: *F* (4.339, 56.40) = 7.724, ***p* = $3.1*10^{-5}$**<br>**Treatment**: *F* (1.315, 17.10) = 7.448, ***p* = 0.0096**<br>**Time x Treatment**: *F* (6.510, 84.62) = 2.497, ***p* = 0.025**<br><br>Vehicle vs CNO<br>All timepoints: *p* > 0.05<br><br>Vehicle vs CNO + fasting<br>4.0 h: *p* = 0.0078<br>4.5 h: *p* = 0.0300<br>All other timepoints: *p* > 0.05 |

| Figure | Sample size [n] | Statistical test | Values |
|---|---|---|---|
| Suppl. 6a | 17 (f:7/m:10) | One-way analysis of covariance (ANCOVA) | **NaCl vs Taltirelin after adjustment**:<br><br>*F* (2.0, 29.0) = 103.77, ***p* $2*10^{-14}$**<br><br>Covariate (body weight): *F* (1.0, 29.0) = 15.40, ***p* $4.8*10^{-4}$** |
| Suppl. 6b | 8 (f:5/m:3) | One-way analysis of covariance (ANCOVA) | **NaCl vs Taltirelin after adjustment**:<br><br>*F* (1.0, 13.0) = 0.10, *p* = 0.754<br><br>Covariate (body weight): *F* (1.0, 13.0) = 14.77, ***p* = 0.002** |
| Suppl. 6c | 5 (f:2/m:3) | One-way analysis of covariance (ANCOVA) | **NaCl vs Taltirelin after adjustment**:<br><br>*F* (1.0, 7.0) = 27.53, ***p* = 0.0012**<br><br>Covariate (body weight): *F* (1.0, 7.0) = 4.44, *p* = 0.073 |
| Suppl. 6d | 16 (f:6/m:10) | REML Mixed-effect model (for missing datapoints) with Greenhouse-Geisser correction | **Time**: *F* (4.12, 61.72) = 15.50, ***p* = $5.9*10^{-9}$**<br>**Treatment**: *F* (1, 15) = 74.00, ***p* = $3.5*10^{-7}$**<br>**Time x Treatment**: *F* (4.01, 54.21) = 18.92, ***p* = $6.9*10^{-10}$**<br><br>NaCl vs Taltirelin |

| | | | |
|---|---|---|---|
| | | followed by<br>Šídák's multiple comparisons test | 4.5 h: *p* = 0.0042<br>5.0 h: *p* < 0.0001<br>5.5 h: *p* < 0.0001<br>6.0 h: *p* < 0.0001<br>6.5 h: *p* = 0.0004<br>7.0 h: *p* = 0.0158<br><br>All other timepoints: *p* > 0.05 |
| Suppl. 6e | 8 (f:5/m:3) | REML Mixed-effect model (for missing datapoints)<br>with<br>Greenhouse-Geisser correction<br><br>followed by<br>Šídák's multiple comparisons test | **Time**: *F* (4.587, 32.11) = 4.414, ***p* = 0.0044**<br>Treatment: *F* (1, 7) = 0.2305, *p* = 0.6458<br>Time x Treatment: *F* (4.611, 27.66) = 0.8851, *p* = 0.4976<br><br>NaCl vs Taltirelin<br><br>All timepoints: *p* > 0.05 |
| Suppl. 6f | 5 (f:2/m:3) | REML Mixed-effect model (for missing datapoints)<br>with<br>Greenhouse-Geisser correction<br><br>followed by<br>Šídák's multiple comparisons test | **Time**: *F* (2.396, 9.584) = 4.625, ***p* = 0.0347**<br>**Treatment**: *F* (1, 4) = 11.5, ***p* = 0.0275**<br>**Time x Treatment**: *F* (1.990, 6.634) = 8.898, ***p* = 0.0133**<br><br>NaCl vs Taltirelin<br>5.5 h: *p* = 0.005<br>6.0 h: *p* = 0.0023<br><br>All other timepoints: *p* > 0.05 |
| Suppl. 6g | *WT:* 16 (f:6/m:10)<br>$Trhr1^{-/-}$: 8 (f:5/m:3)<br>$Trhr2^{-/-}$: 5 (f:2/m:3) | Two-way RM ANOVA<br><br>followed by<br><br>Šídák's multiple comparisons test | **Genotype**: *F* (2, 26) = 9.384, ***p* = 0.0009**<br>**Treatment**: *F* (1, 26) = 62.28, **$p = 2.2*10^{-8}$**<br>**Genotype x Treatment**: *F* (2, 26) = 16.80, **$p = 2.1*10^{-5}$**<br>**Subject**: *F* (26, 26) = 2.964, ***p* = 0.037**<br><br>NaCl vs Taltirelin<br>***WT:* $p = 3.3*10^{-11}$**<br>$Trhr1^{-/-}$: *p* = 0.5362<br>**$Trhr2^{-/-}$: *p* = 0.0002**<br><br>Treatment with Taltirelin<br>***WT* vs. $Trhr1^{-/-}$: $p = 5.3*10^{-8}$**<br>*WT* vs. $Trhr2^{-/-}$: *p* = 0.1107<br>**$Trhr1^{-/-}$ vs. $Trhr2^{-/-}$: *p* = 0.0014**<br><br>All other comparisons: *p* > 0.05 |
| Suppl. 6h | *WT:* 14 (f:5/m:9) | Two-way ANOVA<br>with Greenhouse-Geisser correction<br><br>followed by<br><br>Šídák's multiple comparisons test | **Time**: *F* (2.747, 35.71) = 44.57, **$p = 8.8*10^{-12}$**<br>Treatment: *F* (1, 13) = 2.996, *p* = 0.1071<br>**Interaction**: *F* (2.46, 31.97) = 4.721, ***p* = 0.0113**<br><br>NaCl vs Taltirelin<br>All timepoints: *p* > 0.05 |
| Suppl. 6i | $Trhr1^{-/-}$: 8 (f:5/m:3) | Two-way ANOVA<br>with Greenhouse-Geisser correction<br><br>followed by<br><br>Šídák's multiple comparisons test | **Time**: *F* (1.534, 10.74) = 12.4, ***p* = 0.0025**<br>Treatment: *F* (1, 7) = 0.2995, *p* = 0.6012<br>Interaction: *F* (2.321, 16.25) = 0.2802, *p* = 0.7899<br><br>NaCl vs Taltirelin<br>All timepoints: *p* > 0.05 |
| Suppl. 6j | $Trhr2^{-/-}$: 5 (f:2/m:3) | Two-way ANOVA<br>with Greenhouse-Geisser correction<br><br>followed by<br><br>Šídák's multiple comparisons test | **Time**: *F* (1.745, 5.235) = 10.61, ***p* = 0.0155**<br>Treatment: *F* (1, 4) = 0.00118, *p* = 0.9748<br>Interaction: *F* (1.582, 4.746) = 0.3463, *p* = 0.6783<br><br>NaCl vs Taltirelin<br>All timepoints: *p* > 0.05 |
| Suppl. 6k | *WT:* 14 (f:5/m:9)<br>$Trhr1^{-/-}$: 8 (f:5/m:3) | Two-way ANOVA<br><br>followed by | **Genotype**: *F* (2, 23) = 8.180, ***p* = 0.0021**<br>**Treatment**: *F* (1, 23) = 6.681, ***p* = 0.0167**<br>Interaction: *F* (2, 23) = 0.2075, *p* = 0.8141<br>Subject: *F* (23, 23) = 1.548, *p* = 0.1511 |

| | | | |
|---|---|---|---|
| | *Trhr2*$^{-/-}$: 4<br>(f:1/m:3) | Šídák's multiple comparisons test | <br>NaCl vs Taltirelin<br>***WT*: *p* = 0.0111**<br>*Trhr1*$^{-/-}$: *p* = 0.1961<br>*Trhr2*$^{-/-}$: *p* = 0.3108<br><br>Treatment with NaCl<br>*WT* vs. *Trhr1*$^{-/-}$: *p* = 0.1972<br>*WT* vs. *Trhr2*$^{-/-}$: *p* = 0.2399<br>***Trhr1*$^{-/-}$ vs. *Trhr2*$^{-/-}$: *p* = 0.0146**<br><br>Treatment with Taltirelin<br>***WT* vs. *Trhr1*$^{-/-}$: *p* = 0.0364**<br>*WT* vs. *Trhr2*$^{-/-}$: *p* = 0.5142<br>***Trhr1*$^{-/-}$ vs. *Trhr2*$^{-/-}$: *p* = 0.0112** |
| Suppl. 6l | *WT:* 16<br>(f:6/m:10)<br>*Trhr1*$^{-/-}$: 7<br>(f:4/m:3)<br>*Trhr2*$^{-/-}$: 5<br>(f:2/m:3) | Two-way ANOVA<br><br>followed by<br><br>Šídák's multiple comparisons test | Genotype: *F* (2, 25) = 1.586, *p* = 0.2246<br>**Treatment**: *F* (1, 25) = 13.63, ***p* = 0.0011**<br>Interaction: *F* (2, 25) = 0.3826, *p* = 0.686<br>**Subject**: *F* (25, 25) = 2.071, ***p* = 0.0373**<br><br>NaCl vs Taltirelin<br>***WT:* *p* = 0.0344**<br>***Trhr1*$^{-/-}$: *p* = 0.0342**<br>***Trhr2*$^{-/-}$: *p* = 0.0449**<br><br>Treatment with NaCl<br>*WT* vs. *Trhr1*$^{-/-}$: *p* = 0.6602<br>*WT* vs. *Trhr2*$^{-/-}$: *p* = 0.9602<br>*Trhr1*$^{-/-}$ vs. *Trhr2*$^{-/-}$: *p* = 0.5565<br><br>Treatment with Taltirelin<br>*WT* vs. *Trhr1*$^{-/-}$: *p* = 0.2302<br>*WT* vs. *Trhr2*$^{-/-}$: *p* = 0.9677<br>*Trhr1*$^{-/-}$ vs. *Trhr2*$^{-/-}$: *p* = 0.6849 |
| Suppl. 6m | 16 (f:6/m:10) | Mixed-effects model (REML, for missing datapoints)<br>with Greenhouse-Geisser correction<br><br>followed by<br><br>Šídák's multiple comparisons test | **Time**: *F* (6.511, 97.67) = 47.39, ***p* < $10^{-15}$**<br>**Treatment**: *F* (1, 15) = 4.967, ***p* = 0.0416**<br>**Interaction**: *F* (5.727, 81.13) = 2.302, ***p* = 0.0446**<br><br>NaCl vs Taltirelin<br>All timepoints: *p* > 0.05 |
| Suppl. 6n | 8 (f:5/m:3) | Mixed-effects model (REML, for missing datapoints)<br>with Greenhouse-Geisser correction<br><br>followed by<br><br>Šídák's multiple comparisons test | **Time**: *F* (3.403, 23.82) = 40.04, ***p* < $10^{-15}$**<br>Treatment: *F* (1, 7) = 0.05138, *p* = 0.8272<br>Interaction: *F* (3.916, 25.46) = 1.833, *p* = 0.1545<br><br>NaCl vs Taltirelin<br>All timepoints: *p* > 0.05 |
| Suppl. 6o | 5 (f:2/m:3) | Mixed-effects model (REML, for missing datapoints)<br>with Greenhouse-Geisser correction<br><br>followed by<br><br>Šídák's multiple comparisons test | **Time**: *F* (3.014, 12,06) = 17.62, ***p* = 0.0001**<br>Treatment: *F* (1, 4) = 0.0051, *p* = 0.9467<br>Interaction: *F* (2.408, 8.779) = 0.6454, *p* = 0.5746<br><br>NaCl vs Taltirelin<br>All timepoints: *p* > 0.05 |
| Suppl. 6p | <br>NaCl:<br>*WT:* 9<br>*Trhr1*$^{-/-}$: 8<br>*Trhr2*$^{-/-}$: 16<br><br>Taltirelin:<br>*WT:* 8<br>*Trhr1*$^{-/-}$: 11<br>*Trhr2*$^{-/-}$: 7 | Two-way ANOVA<br><br>followed by<br><br>Šídák's multiple comparisons test | Genotype: *F* (2, 53) = 0.6896, *p* = 0.5062<br>**Treatment**: *F* (1, 53) = 28.41, ***p* = $2*10^{-6}$**<br>**Interaction**: *F* (2, 53) = 5.713, ***p* = 0.0057**<br><br>NaCl vs Taltirelin<br>***WT:* *p* = $6.9*10^{-5}$**<br>*Trhr1*$^{-/-}$: *p* = 0.6206<br>***Trhr2*$^{-/-}$: *p* = 0.0008**<br><br>Treatment with NaCl |

| | | | |
|---|---|---|---|
| | | | *WT* vs. *Trhr1*$^{-/-}$: *p* = 0.3644<br>*WT* vs. *Trhr2*$^{-/-}$: *p* = 0.9564<br>*Trhr1*$^{-/-}$ vs. *Trhr2*$^{-/-}$: *p* = 0.5272<br><br>Treatment with Taltirelin<br>***WT* vs. *Trhr1*$^{-/-}$: *p* = 0.0059**<br>*WT* vs. *Trhr2*$^{-/-}$: *p* = 0.8625<br>*Trhr1*$^{-/-}$ vs. *Trhr2*$^{-/-}$: *p* = 0.099 |
| Suppl. 6q | <u>NaCl:</u><br>*WT:* 13<br>*Trhr1*$^{-/-}$: 8<br>*Trhr2*$^{-/-}$: 20<br><br><u>Taltirelin:</u><br>*WT:* 16<br>*Trhr1*$^{-/-}$: 11<br>*Trhr2*$^{-/-}$: 7 | Two-way ANOVA<br><br>followed by<br><br>Šídák's multiple comparisons test | **Genotype**: *F* (2, 69) = 58.99, ***p* < 10-$^{15}$**<br>Treatment: *F* (1, 69) = 1.382, *p* = 0.2439<br>**Interaction**: *F* (2, 69) = 27.07, ***p* = 2*10$^{-9}$**<br><br>NaCl vs Taltirelin<br>***WT: p* = 5.2*10$^{-10}$**<br>***Trhr1*$^{-/-}$: *p* = 0.0184**<br>*Trhr2*$^{-/-}$: *p* = 0.1124<br><br>Treatment with NaCl<br>***WT* vs. *Trhr1*$^{-/-}$: *p* = 0.0342**<br>***WT* vs. *Trhr2*$^{-/-}$: *p* = 0.0007**<br>***Trhr1*$^{-/-}$ vs. *Trhr2*$^{-/-}$: *p* 1.7*10$^{-7}$**<br><br>Treatment with Taltirelin<br>***WT* vs. *Trhr1*$^{-/-}$: *p* < 10$^{-15}$**<br>***WT* vs. *Trhr2*$^{-/-}$: *p* = 9.5*10$^{-5}$**<br>***Trhr1*$^{-/-}$ vs. *Trhr2*$^{-/-}$: *p* 1.4*10$^{-7}$** |
| Suppl. 6r | <u>NaCl:</u><br>*WT:* 15<br>*Trhr1*$^{-/-}$: 8<br>*Trhr2*$^{-/-}$: 20<br><br><u>Taltirelin:</u><br>*WT:* 16<br>*Trhr1*$^{-/-}$: 11<br>*Trhr2*$^{-/-}$: 7 | Two-way ANOVA<br><br>followed by<br><br>Šídák's multiple comparisons test | **Genotype**: *F* (2, 71) = 17.76, ***p* < 10$^{-15}$**<br>**Treatment**: *F* (1, 71) = 15.82, ***p* = 0.0002**<br>**Interaction**: *F* (2, 71) = 7.472, ***p* = 0.0011**<br><br>NaCl vs Taltirelin<br>***WT: p* = 4.8*10$^{-8}$**<br>*Trhr1*$^{-/-}$: *p* < 0.2572<br>*Trhr2*$^{-/-}$: *p* = 0.6609<br><br>Treatment with NaCl<br>*WT* vs. *Trhr1*$^{-/-}$: *p* = 0.1111<br>*WT* vs. *Trhr2*$^{-/-}$: *p* = 0.1602<br>***Trhr1*$^{-/-}$ vs. *Trhr2*$^{-/-}$: *p* = 0.0015**<br><br>Treatment with Taltirelin<br>***WT* vs. *Trhr1*$^{-/-}$: *p* = 2.6*10$^{-8}$**<br>***WT* vs. *Trhr2*$^{-/-}$: *p* = 0.011**<br>***Trhr1*$^{-/-}$ vs. *Trhr2*$^{-/-}$: *p* = 0.049** |

| Figure | Sample size [n] | Statistical test | Values |
|---|---|---|---|
| Suppl. 7a | 11 (f:4/m:7) | One-way analysis of covariance (ANCOVA) | **Vehicle vs CNO after adjustment:**<br><br>*F* (1.0, 19.0) = 14.39, ***p* < 0.001**<br><br>Covariate (body weight): *F (*1.0, 19.0) = 4.24, *p* = 0.053 |
| Suppl. 7b | 12 (f:6/m:6) | One-way analysis of covariance (ANCOVA) | **Vehicle vs CNO after adjustment:**<br><br>*F* (1.0, 21.0) = 4.88, ***p* = 0.038**<br><br>Covariate (body weight): *F* (1.0, 21.0) = 4.72, ***p* = 0.041** |
| Suppl. 7c | 10 (f:4/m:6) | One-way analysis of covariance (ANCOVA) | **Vehicle vs CNO after adjustment:**<br><br>*F* (1.0, 17.0) = 1.75, *p* = 0.203<br><br>Covariate (body weight): *F* (1.0, 17.0) = 1.78, *p* = 0.200 |

| | | | |
|---|---|---|---|
| Suppl. 7d | Vehicle: 11<br>CNO: 11<br>(f:4/m:7) | Two-way RM ANOVA<br><br>with Greenhouse-Geisser correction<br><br>followed by<br><br>Šídák's multiple comparisons test | **Time**: *F* (5.324, 53.24) = 3.516, ***p* = 0.0070**<br>**Treatment:** *F* (1, 10) = 122, ***p* = 6.3*10$^{-7}$**<br>**Time x Treatment**: *F* (5.031, 50.31) = 5.625, ***p* = 0.0003**<br><br>Vehicle vs CNO<br>3.5 h: *p* = 0.0001<br>4.0 h: *p* = 0.0004<br>4.5 h: *p* < 0.0001<br>5.0 h: *p* = 0.0005<br>5.5 h: *p* = 0.0388<br>6.0 h: *p* = 0.0088<br>7.5 h: *p* = 0.0036<br>8.5 h: *p* = 0.0143<br>All other timepoints: *p* > 0.05 |
| Suppl. 7e | Vehicle: 12<br>CNO: 12<br>(f:6/m:6) | Two-way RM ANOVA<br><br>with Greenhouse-Geisser correction<br><br>followed by<br><br>Šídák's multiple comparisons test | **Time**: *F* (6.576, 72.33) = 2.649, ***p* = 0.019**<br>**Treatment**: *F* (1, 11) = 122, ***p* = 0.002**<br>**Time x Treatment**: *F* (4.817, 52.99) = 1.412, *p* = 0.2365<br><br>Vehicle vs CNO<br>7.0 h: *p* = 0.0216<br>All other timepoints: *p* > 0.05 |
| Suppl. 7f | Vehicle: 9<br>CNO: 9<br>(f:3/m:6) | Two-way RM ANOVA<br><br>with Greenhouse-Geisser correction<br><br>followed by<br><br>Šídák's multiple comparisons test | Time: *F* (4.077, 28.54) = 1.665, *p* = 0.1847<br>**Treatment**: *F* (1, 8) = 17.01, ***p* = 0.0044**<br>**Time x Treatment**: *F* (4.472, 31.30) = 2.699, ***p* = 0.0435**<br><br>Vehicle vs CNO<br>All timepoints: *p* > 0.05 |
| Suppl. 7g | *Trhr1*$^{-/-}$ PVN$^{hM3D}$: 11 (f:4/m:7)<br>*Trhr2*$^{-/-}$ PVN$^{hM3D}$: 12 (f:6/m:6)<br>*Trhr1*$^{-/-}$::*Trhr2*$^{-/-}$ PVN$^{hM3D}$: 8 (f:3/m:5)<br>*WT* PVN$^{hM3D}$: 13 (f:5/m:8)<br>*WT* PVN$^{cherry}$: 14 (f:5/m:9) | Two-way RM ANOVA<br><br>followed by<br><br>Šídák's multiple comparisons test | Genotype: *F* (4, 53) = 1.003, *p* = 0.4142<br>**Treatment**: *F* (1, 53) = 53.05, ***p* 1.6*10$^{-9}$**<br>**Genotype x Treatment**: *F* (4, 53) = 7.267, ***p* = 9.6*10$^{-5}$**<br>**Subject**: *F* (53, 53) = 4,619, ***p* =5.6*10$^{-8}$**<br><br>Vehicle vs CNO<br><br>***Trhr1*$^{-/-}$ PVN$^{hM3D}$: *p* = 1.2*10$^{-5}$**<br>***Trhr2*$^{-/-}$ PVN$^{hM3D}$: *p* = 0.0575**<br>*Trhr1*$^{-/-}$::*Trhr2*$^{-/-}$ PVN$^{hM3D}$: *p* = 0.1886<br>***WT PVN*$^{hM3D}$*: p* = 1*10$^{-7}$**<br>*WT* PVN$^{cherry}$: *p* > 0.9999 |
| Suppl. 7h | 12 (f:5/m:7) | Two-way RM ANOVA<br>with Greenhouse-Geisser correction<br><br>followed by<br><br>Šídák's multiple comparisons test | Time: *F* (4.928, 54.21) = 5.219, *p* = 0.0006<br>Treatment: *F* (1, 11) = 2.128, *p* = 0.1726<br>Time x Treatment: *F* (8.933, 205.4) = 1.367, *p* = 0.2056<br><br>Vehicle vs CNO<br>All other timepoints: *p* > 0.05 |
| Suppl. 7i | 13 (f:6/m:7) | Two-way RM ANOVA<br>with Greenhouse-Geisser correction<br><br>followed by<br><br>Šídák's multiple comparisons test | Time: *F* (5.088, 61.05) = 3.38, *p* = 0.0089<br>Treatment: *F* (1, 12) = 4.720, *p* = 0.0506<br>Time x Treatment: *F* (7.409, 88.91) = 1.103, *p* = 0.2056<br><br>Vehicle vs CNO<br>All other timepoints: *p* > 0.05 |
| Suppl. 7j | 10 (f:4/m:6) | Two-way RM ANOVA<br>with Greenhouse-Geisser correction<br><br>followed by<br><br>Šídák's multiple comparisons test | Time: *F* (4.614, 41.62) = 4.358, *p* = 0.0034<br>Treatment: *F* (1, 9) = 6.67, *p* = 0.0296<br>Time x Treatment: *F* (4.150, 37.35) = 1.862, *p* = 0.1354<br><br>Vehicle vs CNO<br>All other timepoints: *p* > 0.05 |

| Figure | Sample size [n] | Statistical test | Values |
|---|---|---|---|

| | | | |
|---|---|---|---|
| Suppl.<br>8c | 23 °C: 7 (f:3/m:4)<br>10 °C: 5 (f:2/m:3) | Two-tailed paired t test | ***T* (10) = 5.254, *p* = 0.0004** |
| Suppl.<br>8e | 23 °C: 7 (f:3/m:4)<br>10 °C: 6 (f:3/m:3) | Two-tailed paired t test | ***T* (11) = 3.046, *p* = 0.0111** |
| Suppl. 8i | 23 °C: 7 (f:3/m:4)<br>10 °C: 6 (f:3/m:3) | Two-tailed paired t test<br>with Welch´s correction | ***T* (6.641) = 4,961, *p* = 0.0021** |
| Suppl. 8j | Control: 6<br>(f:2/m:4)<br>PVN$^{TRH}$-TeNT: 6<br>(f:2/m:4)<br>DMH$^{TRH}$-TeNT: 7<br>(f:2/m:5)<br>MPA$^{TRH}$-TeNT: 5<br>(f:2/m:3) | One-way ANOVA<br><br>followed by<br><br>Šídák's multiple<br>comparisons test | F (3, 20) = 3.323, ***p* = 0.0406**<br><br>Control vs. PVN$^{TRH}$-TeNT: *p* = 0.6027<br>Control vs. DMH$^{TRH}$-TeNT: *p* = 0.6027<br>Control vs. MPA$^{TRH}$-TeNT: *p* = 0.1667 |
| Suppl.<br>8k | Control: 6<br>(f:2/m:4)<br>PVN$^{TRH}$-TeNT: 6<br>(f:2/m:4)<br>DMH$^{TRH}$-TeNT: 7<br>(f:2/m:5)<br>MPA$^{TRH}$-TeNT: 5<br>(f:2/m:3) | One-way ANOVA | F (3, 20) = 2.547, *p* = 0.0848 |
| Suppl. 8l | Control: 6<br>(f:2/m:4)<br>PVN$^{TRH}$-TeNT: 6<br>(f:2/m:4)<br>DMH$^{TRH}$-TeNT: 7<br>(f:2/m:5)<br>MPA$^{TRH}$-TeNT: 5<br>(f:2/m:3) | One-way ANOVA | F (3, 20) = 1.475, *p* = 0.2515 |
| Suppl.<br>8m | Control: 7<br>(f:3/m:4)<br>PVN$^{TRH}$-TeNT: 7<br>(f:3/m:4)<br>DMH$^{TRH}$-TeNT: 7<br>(f:2/m:5)<br>MPA$^{TRH}$-TeNT: 5<br>(f:2/m:3) | Two-way RM ANOVA<br><br>followed by<br><br>Šídák's multiple<br>comparisons test | **Dark/Light**: *F* (1, 22) = 153.7, ***p* = 2.1*10$^{-11}$**<br>TRH-Population: *F* (3, 22) = 2.421, *p* = 0.0932<br>**Day/night x TRH-Population**:<br>*F* (3, 22) = 12.64, ***p* = 5.1*10$^{-5}$**<br>**Subject**: *F* (22, 22) = 0.7601, *p* = 0.7373<br><br>Dark vs. Light:<br>**Control: *p* = 6.2*10$^{-10}$**<br>**PVN$^{TRH}$-TeNT: *p* = 7.1*10$^{-8}$**<br>DMH$^{TRH}$-TeNT: *p* = 0.0626<br>**MPA$^{TRH}$-TeNT: *p* = 7.2*10$^{-5}$**<br><br>**Dark:**<br>Control vs. PVN$^{TRH}$-TeNT: *p* = 0.0748<br>**Control vs. DMH$^{TRH}$-TeNT: *p* = 1.2*10$^{-6}$**<br>**Control vs. MPA$^{TRH}$-TeNT: *p* = 0.0329**<br>**PVN$^{TRH}$-TeNT vs. DMH$^{TRH}$-TeNT: *p* = 0.0054**<br>PVN$^{TRH}$-TeNT vs. MPA$^{TRH}$-TeNT: *p* = 0.9949<br>DMH$^{TRH}$-TeNT vs. MPA$^{TRH}$-TeNT: *p* = 0.0570<br><br>**Light:**<br>Control vs. PVN$^{TRH}$-TeNT: *p* = 0.999<br>**Control vs. DMH$^{TRH}$-TeNT: *p* = 0.0437**<br>Control vs. MPA$^{TRH}$-TeNT: *p* = 0.5498<br>**PVN$^{TRH}$-TeNT vs. DMH$^{TRH}$-TeNT: *p* = 0.0472**<br>PVN$^{TRH}$-TeNT vs. MPA$^{TRH}$-TeNT: *p* = 0.5696<br>DMH$^{TRH}$-TeNT vs. MPA$^{TRH}$-TeNT: *p* = 0.9050 |
| Suppl.<br>8n | Control: 7<br>(f:3/m:4)<br>PVN$^{TRH}$-TeNT: 7<br>(f:3/m:4)<br>DMH$^{TRH}$-TeNT: 7<br>(f:2/m:5)<br>MPA$^{TRH}$-TeNT: 5<br>(f:2/m:3) | One-way ANOVA | F (3, 22) = 2.421, *p* = 0.0932 |
| Suppl.<br>8o | 23°C Control: 14<br>(f:7/m:7)<br>Control: 7 | One-way ANOVA | F (4, 35) = 1.326, *p* = 0.2797 |

| | | | |
|---|---|---|---|
| | (f:3/m:4)<br>PVN$^{TRH}$-TeNT: 7<br>(f:3/m:4)<br>DMH$^{TRH}$-TeNT: 7<br>(f:2/m:5)<br>MPA$^{TRH}$-TeNT: 5<br>(f:2/m:3) | | |
| Suppl.<br>8p | 23°C Control: 16<br>(f:7/m:9)<br>Control: 7<br>(f:3/m:4)<br>PVN$^{TRH}$-TeNT: 7<br>(f:3/m:4)<br>DMH$^{TRH}$-TeNT: 7<br>(f:2/m:5)<br>MPA$^{TRH}$-TeNT: 5<br>(f:2/m:3) | One-way ANOVA<br><br>followed by<br><br>Tukey´s multiple comparisons test | F (4, 37) = 21.03, ***p* = 4.1*10$^{-9}$**<br><br>**Control 23°C vs. Control 10°C: *p* = 0.0013**<br>**Control 23°C vs. PVN$^{TRH}$-TeNT 10°C: *p* = 6.0*10$^{-8}$**<br>**Control 23°C vs. DMH$^{TRH}$-TeNT 10°C: *p* = 1.3*10$^{-7}$**<br>**Control 23°C vs. DMH$^{TRH}$-TeNT: *p* = 0.0093**<br><br>All other comparisons: p > 0.05 |

| Figure | Sample size [n] | Statistical test | Values |
|---|---|---|---|
| Suppl.<br>9b | Control: 11<br>(f:5/m:6)<br>PVN$^{TRH}$-hM3D:11<br>(f:5/m:6)<br>DMH$^{TRH}$-hM3D:10<br>(f:4/m:6)<br>MPA$^{TRH}$-hM3D:11<br>(f:5/m:6) | One-way ANOVA test | *F* (3, 39) = 1.694, *p* = 0.1842 |
| Suppl.<br>9c | Control: 11<br>(f:5/m:6)<br>PVN$^{TRH}$-hM3D:11<br>(f:5/m:6)<br>DMH$^{TRH}$-hM3D:10<br>(f:4/m:6)<br>MPA$^{TRH}$-hM3D:11<br>(f:5/m:6) | One-way ANOVA test | *F* (3, 39) = 0.8831, *p* = 0.5555 |
| Suppl.<br>9d | Control: 11<br>(f:5/m:6)<br>PVN$^{TRH}$-hM3D:11<br>(f:5/m:6)<br>DMH$^{TRH}$-hM3D:10<br>(f:4/m:6)<br>MPA$^{TRH}$-hM3D:11<br>(f:5/m:6) | One-way ANOVA test | *F* (3, 39) = 0.5537, *p* = 0.6487 |
| Suppl.<br>9e | Control: 12<br>(f:6/m:6)<br>PVN$^{TRH}$-hM3D:12<br>(f:6/m:6)<br>DMH$^{TRH}$-hM3D:12<br>(f:6/m:6)<br>MPA$^{TRH}$-hM3D:12<br>(f:6/m:6) | One-way ANOVA test<br><br>followed by<br><br>Tukey's multiple comparisons test | *F* (3, 44) = 12.10, ***p* = 6.6*10$^{-6}$**<br><br>Control vs. PVN$^{TRH}$-hM3D: *p* = 6.3*10$^{-6}$<br>Control vs. DMH$^{TRH}$-hM3D: *p* = 0.0103<br>Control vs. MPA$^{TRH}$-hM3D: *p* = 0.00018 |

**Supplementary Table 2. Primary antibodies**

| Target | Type | Clonality | Host | Dilution | Company | Ab Code |
|---|---|---|---|---|---|---|
| **cFos** | Primary | Monoclonal | Rabbit | 1:500 | Cell Signaling | 9F6 |
| **GFP** | Primary | Polyclonal | Chicken | 1:1000 | Abcam | ab13970 |
| **mCherry** | Primary | Polyclonal | Goat | 1:1000 | OriGene | AB0040-500 |
| **Tyrosine Hydroxylase** | Primary | Polyclonal | Rabbit | 1:500 | Merck | AB152 |
| **Tryptophane-hydroxylase 2** | Primary | Polyclonal | Rabbit | 1:500 | Novus Biological | NB100-74555 |
| **pHSL [Ser660]** | Primary | Polyclonal | Rabbit | 1:5000 | Cell Signaling | 4126S |
| **HSL** | Primary | Polyclonal | Rabbit | 1:5000 | Cell Signaling | 4107S |
| **alpha-Tubulin** | Primary | Monoclonal | Mouse | 1:1000 | Merck, Sigma Aldrich | T6793 |

**Supplementary Table 3. Secondary antibodies**

| Target | Type | Clonality | Host | Dilution | Company | Ab Code |
|---|---|---|---|---|---|---|
| **Alexa Fluor® 488 AffiniPure™ Donkey Anti-Chicken** | Secondary | Polyclonal | Donkey | 1:1000 | Jackson Immuno | AB_2340375 |
| **Cy™3 AffiniPure™ Donkey Anti-Goat IgG (H+L)** | Secondary | Polyclonal | Donkey | 1:1000 | Jackson Immuno | AB_2307351 |
| **Donkey anti-Rabbit IgG (H+L) Highly Cross-Adsorbed Secondary Antibody, Alexa Fluor™ 488** | Secondary | Polyclonal | Donkey | 1:1000 | Invitrogen | A-21206 |
| **Donkey anti-Rabbit IgG (H+L) Highly Cross-Adsorbed Secondary Antibody, Alexa Fluor™ 555** | Secondary | Polyclonal | Donkey | 1:1000 | Invitrogen | A-31572 |
| **Donkey anti-Rabbit IgG (H+L) Highly Cross-Adsorbed Secondary Antibody, Alexa Fluor™ 647** | Secondary | Polyclonal | Donkey | 1:1000 | Invitrogen | A31573 |
| **Goat Anti-Rabbit IgG -HRP Conjugate** | Secondary | Polyclonal | Goat | 1:2500 | Agilent | P0448 |
| **Polyclonal Goat Anti-Mouse Immunoglobulins /HRP.** | Secondary | Polyclonal | Goat | 1:2500 | Agilent | P0447 |

**Supplementary Table 4. RNAScope Probes**

| Probe | Catalog number | Lot Nummer | Channel | Company |
|---|---|---|---|---|
| **Mm-*Trh*** | 436811-C2 | 22238B | C2 | ACD Bio-Techne |
| **Mm-*Trhr1*** | 443771-C3 | 22238B | C3 | ACD Bio-Techne |
| **Mm-*Trhr2*** | 1115121-C1 | 21280A | C1 | ACD Bio-Techne |
| **Mm-*Fos*** | 316921-C1/-C2 | 24355A/24277A | C1/C2 | ACD Bio-Techne |
| **Mm-*Agrp*** | 400711-C3 | 22070A | C3 | ACD Bio-Techne |

## *References:*

1. Allen Reference Atlas – Mouse Brain [Adult Mouse]. Available from atlas.brain-map.org.
