# Supplementary material for: Thyrotropin-releasing hormone neurons of different hypothalamic nuclei increase energy expenditure
Source: Nat Commun. 2026 Apr 15;17:3499. doi: 10.1038/s41467-026-71617-3 (PMC13083850; doi:10.1038/s41467-026-71617-3)
Supplement: Supplementary file 2 — Reporting Summary [file 41467_2026_71617_MOESM2_ESM.pdf]

Corresponding author(s): Helge Müller-Fielitz

Last updated by author(s): Mar 6, 2026

Reporting Summary

Nature Portfolio wishes to improve the reproducibility of the work that we publish. This form provides structure for consistency and transparency in reporting. For further information on Nature Portfolio policies, see our Editorial Policies and the Editorial Policy Checklist.

Statistics

For all statistical analyses, confirm that the following items are present in the figure legend, table legend, main text, or Methods section.

n/a	Confirmed	
☐	☒	The exact sample size (*n*) for each experimental group/condition, given as a discrete number and unit of measurement
☐	☒	A statement on whether measurements were taken from distinct samples or whether the same sample was measured repeatedly
☐	☒	The statistical test(s) used AND whether they are one- or two-sided<br>*Only common tests should be described solely by name; describe more complex techniques in the Methods section.*
☐	☒	A description of all covariates tested
☐	☒	A description of any assumptions or corrections, such as tests of normality and adjustment for multiple comparisons
☐	☒	A full description of the statistical parameters including central tendency (e.g. means) or other basic estimates (e.g. regression coefficient) AND variation (e.g. standard deviation) or associated estimates of uncertainty (e.g. confidence intervals)
☐	☒	For null hypothesis testing, the test statistic (e.g. *F*, *t*, *r*) with confidence intervals, effect sizes, degrees of freedom and *P* value noted<br>*Give P values as exact values whenever suitable.*
☒	☐	For Bayesian analysis, information on the choice of priors and Markov chain Monte Carlo settings
☒	☐	For hierarchical and complex designs, identification of the appropriate level for tests and full reporting of outcomes
☒	☐	Estimates of effect sizes (e.g. Cohen's *d*, Pearson's *r*), indicating how they were calculated

Our web collection on statistics for biologists contains articles on many of the points above.

Software and code

Policy information about availability of computer code

Data collection	Indirect Calorometry; TSE PhenoMaster,<br>DSI Telemetri setup; TA-F10 Transmitter for temperature and activity; Data Science International; Analysed with Software: Dataquest A.R.T.™ 4.3<br>Infrared thermography; VarioCam® head HiRes 640 (InfraTec); Analysed with IRBIS® 3<br>Western Blot, Detector: Fusio Solo S Vilber; analysed with EvolutionCapt 17.0.1 (Vilber Lourmat)<br>qPCR: LightCycler® 96 (Roche); Version 1.1.0.1320<br>Picture Analysis: Fiji, ImageJ 1.52i<br>Image aquisition; Leica Stelaris 5; Leica SP5,<br>Adobe Ilustrator CS5<br>BioRender
Data analysis	We use Graphpad 10.4 for data and statistical analysis<br>Picture Analysis: Fiji, ImageJ 1.52i

For manuscripts utilizing custom algorithms or software that are central to the research but not yet described in published literature, software must be made available to editors and reviewers. We strongly encourage code deposition in a community repository (e.g. GitHub). See the Nature Portfolio guidelines for submitting code & software for further information.

Data

Policy information about availability of data

All manuscripts must include a data availability statement. This statement should provide the following information, where applicable:

- Accession codes, unique identifiers, or web links for publicly available datasets
- A description of any restrictions on data availability
- For clinical datasets or third party data, please ensure that the statement adheres to our policy

All data are available in the article and its Supplementary files or from the corresponding author upon request. Source data are provided with this paper.

Research involving human participants, their data, or biological material

Policy information about studies with human participants or human data. See also policy information about sex, gender (identity/presentation), and sexual orientation and race, ethnicity and racism.

Reporting on sex and gender	Not applicable
Reporting on race, ethnicity, or other socially relevant groupings	Not applicable
Population characteristics	Not applicable
Recruitment	Not applicable
Ethics oversight	Not applicable

Note that full information on the approval of the study protocol must also be provided in the manuscript.

Field-specific reporting

Please select the one below that is the best fit for your research. If you are not sure, read the appropriate sections before making your selection.

[x] Life sciences [ ] Behavioural & social sciences [ ] Ecological, evolutionary & environmental sciences

For a reference copy of the document with all sections, see nature.com/documents/nr-reporting-summary-flat.pdf

Life sciences study design

All studies must disclose on these points even when the disclosure is negative.

Sample size	We conducted a sample size calculation as required by the ethical authorities, although no specific sample size calculation was performed for each individual experiment. The sample size for each experiment was determined based on standard practices in rodent research and informed by previous studies.
Data exclusions	Sampels or animals were only excluded in the event of technical errors (defective sensors, faulty extraction, loss of sample)
Replication	All experiments used littermate mice from at least 3-4 different cohorts to ensure sufficient genetic and physiological variability throughout the experiments. Due to the capacity of the calorimetric measurements, each cohort consisted of 8 animals. No significant differences were observed between cohorts in the data analysis. All attempts at replicates were successful.
Randomization	For treatment allocation, mice were randomized. Chemogenetic experiments were designed using a cross-over experimental design, ensuring that each mouse received both the chemogenetic actuator (clozapine-N_oxide, CNO) and the vehicle (saline solution).
Blinding	Whenever possible experimenters were blinded for AAV genotype, sampel loading and treatment.

Reporting for specific materials, systems and methods

We require information from authors about some types of materials, experimental systems and methods used in many studies. Here, indicate whether each material, system or method listed is relevant to your study. If you are not sure if a list item applies to your research, read the appropriate section before selecting a response.

Materials & experimental systems

n/a	Involved in the study
	☒ Antibodies
☒	Eukaryotic cell lines
☒	Palaeontology and archaeology
	☒ Animals and other organisms
☒	Clinical data
☒	Dual use research of concern
☒	Plants

Methods

n/a	Involved in the study
☒	ChIP-seq
☒	Flow cytometry
☒	MRI-based neuroimaging

Antibodies

Antibodies used

The list of antibodies can be found in the extended tables 2 and 3.

Rabbit c fos; Cell Signaling; # 9F6;
Goat mCherry; OriGene; # AB0040-500;
chicken GFP; AbCam; # ab13970;
Rabbit TH; Merck; # AB152;
Rabbit TPH; Novus Biological; #NB100-74555
Rabbit Oxytocin; ImmmunoStar; #20068
Rabbit pHSL [Ser660]; Cell Signaling; #4126S
Rabbit HSL; Cell Signaling; #4107S
mouse alpa-Tubulin; Merck, Sigma Aldrich; # T6793
Alexa Fluor® 488 AffiniPure™ Donkey Anti-Chicken, Jackson Immuno, #AB_2340375
Cy™3 AffiniPure™ Donkey Anti-Goat IgG (H+L), ackson Immuno, #AB_2307351
Donkey anti-Rabbit IgG (H+L) Highly Cross-Adsorbed Secondary Antibody, Alexa Fluor™ 488; Invitrogen; #A-21206
Donkey anti-Rabbit IgG (H+L) Highly Cross-Adsorbed Secondary Antibody, Alexa Fluor™ 555; Invitogen; #A-31572
Donkey anti-Rabbit IgG (H+L) Highly Cross-Adsorbed Secondary Antibody, Alexa Fluor™ 647; Invitrogen; #A31573
Goat Anti-Rabbit IgG -HRP Conjugate; Agilent; #P0448
Polyclonal Goat Anti-Mouse Immunoglobulins/HRP; Agilent; #P0447

Validation

All antibodies were validated through the histochemical analysis conducted by the investigators at the Institute of Experimental and Clinical Pharmacology and Toxicology in Lübeck. Additionally, the antibodies were validated by the manufacturing company.

Rabbit c fos; Cell Signaling; https://www.cellsignal.com/products/primary-antibodies/c-fos-9f6-rabbit-mab/2250
Goat mCherry; OriGene; https://www.origene.com/catalog/antibodies/tag-antibodies/ab0040-500/mcherry-goat-polyclonal-antibody
chicken GFP; AbCam; https://www.abcam.com/en-us/products/primary-antibodies/gfp-antibody-ab13970
Rabbit TH; Merck Sigma Aldrich; https://www.sigmaaldrich.com/DE/de/product/mm/ab152
Rabbit TPH; Novus Biological; #NB100-74555; https://www.novusbio.com/products/tryptophan-hydroxylase-2-antibody_nb100-74555
Rabbit Oxytocin; ImmmunoStar; #20068; https://www.immunostar.com/product/oxytocin-antibody/
Rabbit pHSL [Ser660]; Cell Signaling; #4126S; https://www.cellsignal.com/products/primary-antibodies/phospho-hsl-ser660-antibody/4126
Rabbit HSL; Cell Signaling; #4107S; https://www.cellsignal.com/products/primary-antibodies/hsl-antibody/4107?utm_term&utm_campaign=SO-Products-PrimaryAntibodies&utm_source=adwords&utm_medium=ppc&hsa_acc=8625036580&hsa_cam=21865491299&hsa_grp=167649643577&hsa_ad=719485262778&hsa_src=g&hsa_tgt=dsa-2373170399499&hsa_kw&hsa_mt&hsa_net=adwords&hsa_ver=3&gclid=Cj0KCQiA_9u5BhCUARIsABbMSPuYIr_notB97bYMIUww9uAEQv2CFyCNEFKHzzXTfGdGOnteTPIg47EaAsrTEALw_wcB
mouse alpa-Tubulin; Merck, Sigma Aldrich; # T6793; https://www.sigmaaldrich.com/DE/de/product/sigma/t6793
utm_source=google&utm_medium=cpc&utm_id=12479849269&utm_campaign=%7Bcampaignname%7D&utm_content=118951894299&utm_term=&gclid=Cj0KCQiA_9u5BhCUARIsABbMSPt4OMi_QaxRl5T6EDVO4ciQtMGBCOF1zkd06Bbcf4P2gqYo8165hDoaAhX8EALw_wcB

Animals and other research organisms

Policy information about studies involving animals; ARRIVE guidelines recommended for reporting animal research, and Sex and Gender in Research

Laboratory animals

Mice were group housed (3-5 animals per cage) in a controlled environment regarding to humidity and temperature (22-24℃) on a 12 h light/12 h dark cycle. Mice had ad libitum access to water and to a standard rodent chow diet.
All mouse lines were established on a C57BL/6N background. The mouse lines used were described previously: TRH-IRES-Cre+/- (Trhtm1.1(cre)Mjkr), PMID: 24487620; Trhr1-/-, Trhrtm1Bau, PMID: 14988432, and Trhr2-/-, obtained from Deltagen, PMID: 28883467. Trhr1-/- and Trhr2-/- were bred together to generate Trhr1-/-:Trhr2-/- double knockout mice.

Wild animals	Not applicable
Reporting on sex	Both sexes were used. No sex differences were tested.
Field-collected samples	Not applicable
Ethics oversight	All animal experiments were conducted in accordance with the protocols approved by the local government authorities (Ministerium für Landwirtschaft, ländliche Räume, Europa und Verbraucherschutz, Kiel, Germany) and were performed in agreement with the Rules of Laboratory Animal Care and International Law on Animal Experimentation.

Note that full information on the approval of the study protocol must also be provided in the manuscript.

Plants

Seed stocks	Not applicable
Novel plant genotypes	Not applicable
Authentication	Not applicable
